# Genetic Diversity and Structure of *Physaria* on the Kaibab Plateau: Implications for Conservation

**DOI:** 10.1002/ece3.70523

**Published:** 2024-11-18

**Authors:** Jer Pin Chong, Jamie Minnaert‐Grote, David N. Zaya, Mary V. Ashley, Janice Coons, Jennifer M. Ramp Neale, Brenda Molano‐Flores

**Affiliations:** ^1^ Department of Biological Sciences University of Illinois Chicago Illinois USA; ^2^ Illinois Natural History Survey, Prairie Research Institute University of Illinois Urbana‐Champaign Champaign Illinois USA; ^3^ Biological Sciences Department Eastern Illinois University Charleston Illinois USA; ^4^ Department of Research & Conservation Denver Botanic Gardens Denver Colorado USA

**Keywords:** conservation, genetic diversity, Kaibab Plateau, *Physaria*

## Abstract

Assessing patterns of genetic diversity within and among closely related congeners is important for evaluating the conservation status of rare plant taxa. We used nuclear microsatellite genotyping to examine the genetic relatedness among three *Physaria* taxa on the Kaibab Plateau in northern Arizona. Over 100 species of *Physaria* are recognized, and are widely distributed in Western North America. On the Kaibab Plateau several taxa occur, including *Physaria arizonica* and two subspecies of *P. kingii, P. kingii* subsp. *latifolia* and *P. kingii* subsp. *kaibabensis*. The latter subspecies, the Kaibab bladderpod, is rare and endemic to the Kaibab Plateau and is a potential candidate to be listed under the Endangered Species Act. Morphological characters, primarily flower color, have been used to distinguish *P. kingii* subsp. *kaibabensis* from other subspecies. Here we aim to assess its genetic diversity and differentiation as compared to congeners on and around the Kaibab Plateau. We genotyped DNA obtained from leaf samples from 463 individuals collected from 26 sites representing the three putative taxa (12 *P. kingii* subsp. *kaibabensis*, 8 *P. kingii* subsp. *latifolia*, and 6 *P. arizonica*). Our results showed that all samples initially identified as *P. kingii* subsp. *latifolia* and *P. kingii* subsp. *kaibabensis* on the Kaibab Plateau form a single genetic cluster that is well‐differentiated from *P. kingii* subsp. *latifolia* sampled from sites off the plateau or *P. arizonica* on or off the plateau. For *P. kingii* on the plateau, our findings do not support the previous subspecies designations based on morphological characters. While additional studies of *P. kingii* will further resolve taxonomic uncertainties within this species, our findings indicate that the Kaibab Plateau population is genetically diverse and genetically distinct, and federal protection is justified in light of the threats faced on the Kaibab Plateau and its limited range.

## Graphical abstract

We conducted a population genetics study on three plant taxa in the genus *Physaria* (*P. kingii* subsp. *kaibabensis*, *P. kingii* subsp. *latifolia*, and *P. arizonica*) that are found on the Kaibab Plateau, Arizona, USA. For *P. kingii* on the Kaibab Plateau, our findings do not support the previous subspecies designations based on morphological characters, but the Kaibab Plateau population of *P. kingii* is genetically diverse and genetically distinct.

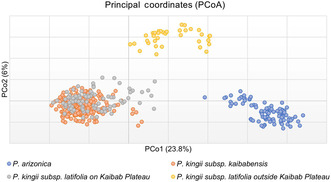

## 1 Introduction

Integrating genetic information in decision‐making for threatened plants has become fundamental to conservation practice. Understanding genetic relationships among populations of rare species is vital in designating conservation priorities that maintain or restore genetic diversity (DeMauro 1993; Kim et al. 2015; Tecic et al. 1998; Whiteley et al. 2015; Zaya et al. 2017), detecting populations with low diversity (Backs, Terry, and Ashley 2016; Finger et al. 2011), and delimiting genetic management units (Bradbury et al. 2023; Zhou et al. 2010). Once the genetic diversity of rare species is well described, conservation management may aim to establish intermediate populations to promote genetic connectivity among isolated populations, reduce impacts of inbreeding and genetic drift, and increase adaptive potential and species resilience (Hanson, Fuller, and Rhodes 2019; Kelly and Phillips 2016; Tkach and Watson 2023).

This study investigates the population genetic structure among three *Physaria* taxa on the Kaibab Plateau, northern Arizona, USA. The Kaibab Plateau is the southernmost plateau of a series of high plateaus that extend north through Utah. It is a “sky island,” with approximately 2980 sq. km above 1800 m elevation, bounded on all sides by escarpments and steep slopes. The vegetation is dominated by spruce‐fir and ponderosa pine forests characteristic of more northern latitudes, and the plateau is separated from similar habitats by surrounding deserts (Rasmussen 1941; Rink, Hodgson, and Phillips 2020).

**FIGURE 1:**
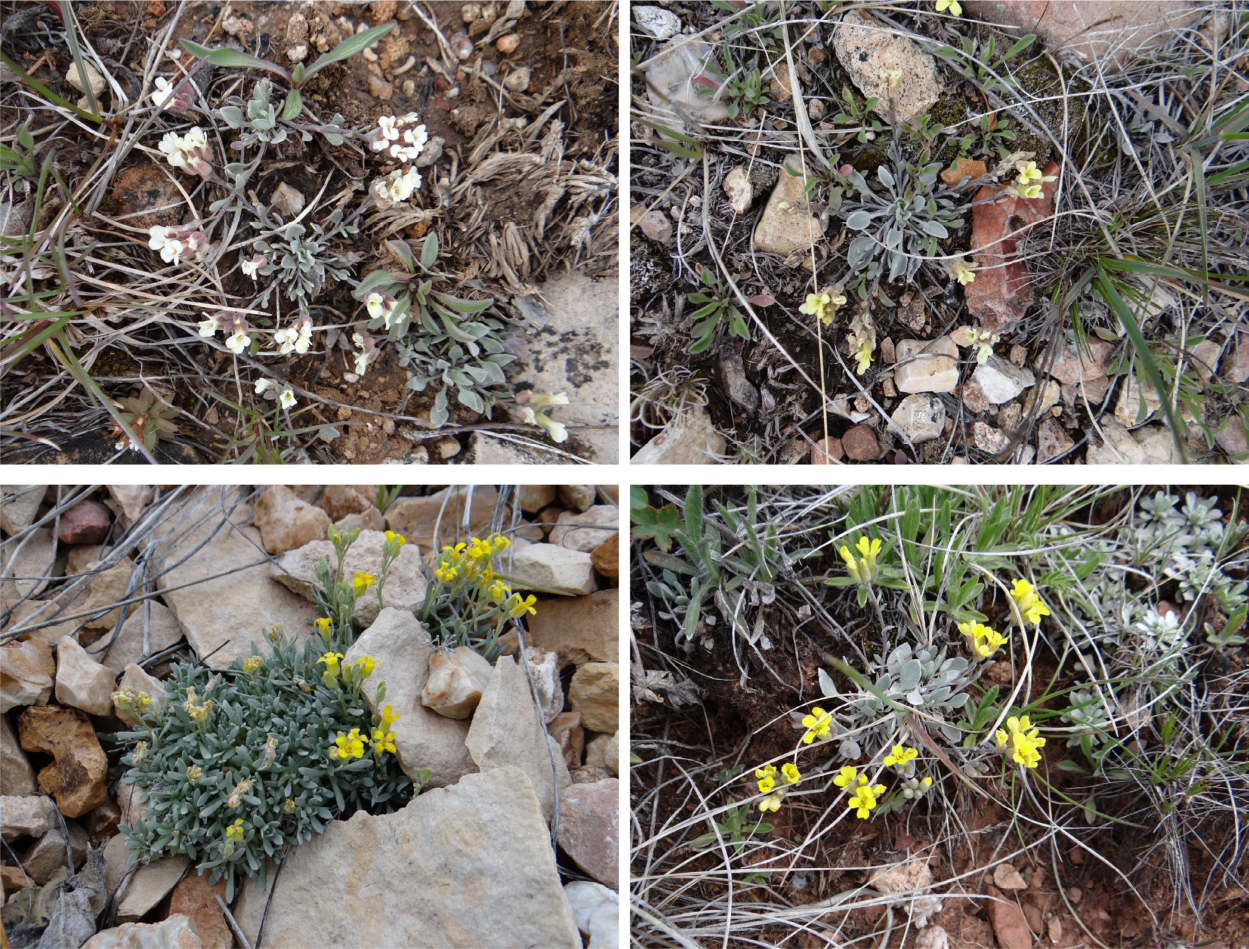
Images of the *Physaria kingii* subsp. *kaibabensis* and their congeners in bloom, observed on the Kaibab plateau. Top left: *P. kingii* subsp. *kaibabensis* with white inflorescences. Top right: *P. kingii* subsp. *kaibabensis* with more yellow inflorescences. Bottom left: *P. arizonica*. Bottom right: *P. kingii* subsp. *latifolia*.

Because of the isolation of the Kaibab Plateau, seed and pollen dispersal (and thus gene flow) is limited for many plants that occur there. Several plant species are endemic to the Kaibab Plateau, and two, *Castilleja kaibabensis* N.H. Holmgren (Kaibab paintbrush) and *Physaria kingii* subsp. *kaibabensis* (Rollins) O'Kane (Kaibab bladderpod, Figure 1) are of conservation concern (Rink, Hodgson, and Phillips 2020). Our focus was on *P kingii* subsp. *kaibabensis* and two congeners that co‐occur on the plateau, *P. kingii* subsp. *latifolia* (A. Nelson) O'Kane & Al‐Shehbaz (King bladderpod), and *Physaria arizonica* (S. Watson) O'Kane & Al‐Shehbaz (Arizona bladderpod). Members of *Physaria* (Brassicaceae) are commonly known as twinpods or bladderpods because of their globose or inflated fruits (silicles). Currently, 106 *Physaria* species occur in North America, South America, and Asia, with their center of endemism in Western North America (Al‐Shehbaz 2012). They often have a decumbent growth form and racemes of yellow flowers. They can be annual, biennial, or perennial; our study species are perennial forbs. *P. arizonica* and *P. kingii* subsp. *latifolia* have wide distributions in western North America. *P. arizonica* is found in northern Arizona and southern Utah, whereas *P. kingii* subsp. *latifolia* is found in northern Arizona, eastern California, southern Nevada, and southern Utah (O'Kane 2010).

These three *Physaria* taxa differ in growth forms and/or flower colors. *P. arizonica* produces vibrant yellow flowers and mostly erect stems. *P. kingii* flowers can range in color from cream to yellow and stems that are prostate or decumbent. *P. kingii* subsp. *latifolia* and *P. kingii* subsp. *kaibabensis* reportedly have subtle differences in flower coloration and reproductive structures. *P. kingii* subsp. *kaibabensis* (Figure 1) has cream‐white or white flowers with styles 0.5–2.5 mm long and six to eight ovules per ovary (Minnaert‐Grote 2014; O'Kane 2010). *P. kingii* subsp. *latifolia* has more ovules per ovary (8–16) and longer styles (7 mm long), although *P. kingii* subsp. *latifolia* on the Kaibab Plateau have shorter styles, similar to *P. kingii* subsp. *kaibabensis* (Minnaert‐Grote 2014). Flowers of *P. kingii* subsp. *latifolia* are typically yellow, but some plants on the Kaibab Plateau have more cream or white flowers (O'Kane 2010). Confusion arises because *P. kingii* subsp. *latifolia* on the plateau has alternatively been described as a hybrid (*P. kingii* subsp. *kaibabensis* x *P. arizonica*) that is more similar to *P. wardii* (Minnaert‐Grote 2014). *P. kingii* subsp. *kaibabensis* and *P. kingii* subsp. *latifolia* occur on the central and eastern parts of the plateau while *P. arizonica* is primarily distributed on the northern part of the plateau, but there are no obvious physical or ecological barriers isolating populations on the plateau. There are also no phenological barriers to hybridization because these *Physaria* taxa all bloom from May to June (Minnaert‐Grote 2014; O'Kane 2010).

*Physaria kingii* subsp. *kaibabensis* is currently listed as G5T3Q and S3 in Arizona, a taxon that is vulnerable with a moderate risk of extinction (Arizona Game and Fish Department 2024; NatureServe 2024). US Forest Service and the state of Arizona listed *P. kingii* subsp. *kaibabensis* as a sensitive taxon considering it is only found on the Kaibab Plateau (Arizona Game and Fish Department 2024). In 2009, United States Fish and Wildlife Service (USFWS) made an effort to list *P. kingii* subsp. *kaibabensis* under the Endangered Species Act in the southwestern US (United States Fish and Wildlife Service 2009) and as of 2024 the status of this species has not changed (NatureServe 2024). The *P. kingii* subsp. *kaibabensis* populations are threatened by habitat destruction, pre‐dispersal seed predation, disturbance by ungulates and gophers or other rodents, hybridization, and climate change (Molano‐Flores and Coons 2020). The Land and Resource Management Plan for the Kaibab National Forest (USDA. United States Department of Agriculture 2014) has provided management recommendations for the habitats where *P. kingii* subsp. *kaibabensis* is found. However, no specific conservation strategies have been implemented to address the risks of hybridization.

Systematics of *P. kingii* is historically complex and it has been a challenging group to delimit (Al‐Shehbaz and O'Kane 2002; Holmgren 2004; O'Kane 2007). Currently, seven subspecies of *P. kingii* are recognized and distributed across Western North America. A previous genetic study attempting to resolve taxonomic uncertainties within *P. kingii* using nuclear and chloroplast sequences found that *P. kingii* subsp. *kaibabensis* is closely related to *P. kingii* subsp. *latifolia* on the Kaibab Plateau, but generally not as closely related to *P. kingii* subsp. *latifolia* in other regions (Minnaert‐Grote 2014). Additionally, *P. kingii* subsp. *kaibabensis* was closely related to *P. arizonica* in a phylogenetic tree constructed by nuclear ITS marker, but not in the *rps* intron and *ndh*C‐*trn*V intergenic spacer chloroplast tree (Minnaert‐Grote 2014). These findings led the author to speculate that *P. kingii* subsp. *latifolia* on the Kaibab Plateau resulted from hybridization between *P. kingii* subsp. *kaibabensis* and *P. arizonica*. While chromosome studies of *P. kingii* have reported diploid counts (Harley, Rollins, and Shaw 1974; Rollins and Rüdenberg 1977), a more recent study reported that while *P. kingii* subsp. *kaibabensis* is diploid; *P. kingii* subsp. *latifolia* collected from a site on the edge of the Kaibab Plateau was tetraploid (Salywon, Rebman, and Dierig 2022). A broader survey of chromosome counts is needed to determine if polyploidy is characteristic of *P. kingii* subsp. *latifolia* individuals on the Kaibab Plateau. If so, this could lead to genetic isolation of the two subspecies. It was unknown whether Salywon, Rebman, and Dierig (2022) found an autopolyploid or allopolyploid; if it were the latter, it would lend support for the hybrid classification suggested by Minnaert‐Grote (2014).

The main objective of this study was to determine if *P. arizonica, P. kingii* subsp. *kaibabensis*, and *P. kingii* subsp. *latifolia* are genetically differentiated in a way that corresponds to the current taxonomic units designated based on morphological characters. We collected samples on and off the Kaibab Plateau of northern Arizona for microsatellite genotyping. We compared genetic clusters to morphological assignments to species and subspecies to test our hypothesis that three genetic clusters would be found and correspond to the morphological identification of the three *Physaria* taxa. We aimed to improve our understanding of the conservation status of *P. kingii* subsp. *kaibabensis*, assess whether it is threatened by gene flow with congeners, and contribute to the discussion of whether *P. kingii* subsp. *kaibabensis* warrants legal protection status.

## 2 Materials and Methods

### 2.1 Sampling Collection

**FIGURE 2:**
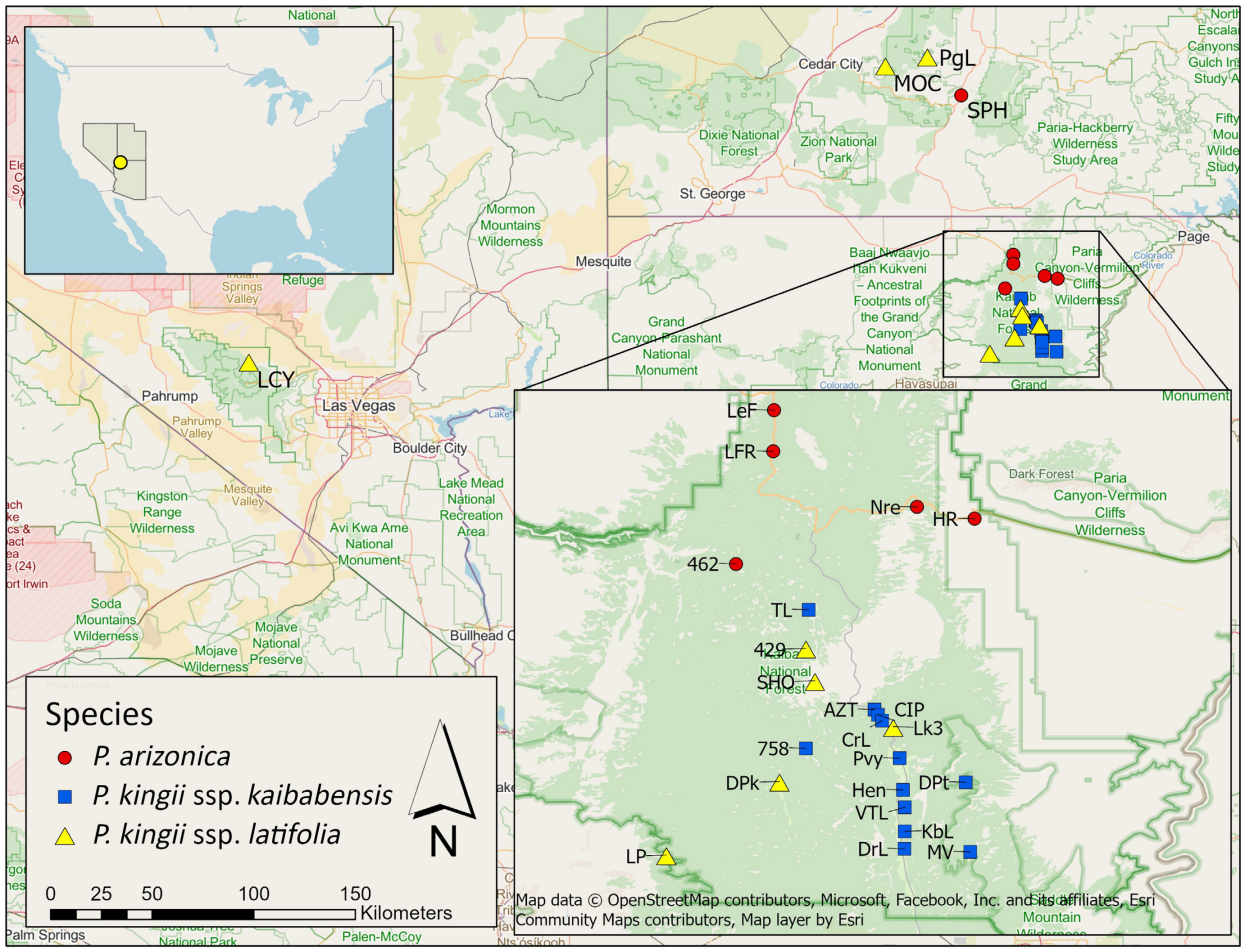
Map of the 26 *Physaria* sampling sites on and nearby the Kaibab Plateau of northern Arizona. *P. arizonica* populations are shown in red circles, *P. kingii* subsp. *kaibabensis* populations in blue squares, and *P. kingii* subsp. *latifolia* populations in yellow triangles. The basemap data were provided by OpenStreetMap (https://www.openstreetmap.org/copyright).

We sampled leaves from 10 to 20 individual *Physaria* from each of 26 locations on and near the Kaibab Plateau of northern Arizona, for a total of 463 samples (Figure 2). Each plant was identified to species or subspecies based on morphological characters according to Minnaert‐Grote (2014). A total of 94 *P. arizonica* samples were collected from six sites, 240 *P. kingii* subsp. *kaibabensis* samples were collected from 12 sites and 129 *P. kingii* subsp. *latifolia* samples were collected from eight sites (Table 1). Leaf samples from each plant were put in individual envelopes and stored in Ziploc bags with silica to dry and preserve the leaf tissues. For each plant sample, genomic DNA was extracted using DNeasy Plant Mini Kit (Qiagen).

**TABLE 1:** Summary of *Physaria* sampling. A total of 463 *Physaria* leaf samples from 26 locations (10–20 from each sampling site) were collected and genotyped. Species and subspecies designations were based on morphological characteristics and elements of occurrence records.

Population	Species	Location	Sample genotyped
462	*P. arizonica*	NF 461 & NF462	20
HR	*P. arizonica*	House Rock Viewpoint	20
LeF	*P. arizonica*	Le Fevre Overlook	20
LFR	*P. arizonica*	Le Fevre Ridge	10
Nre	*P. arizonica*	North Rim East	12
SPH	*P. arizonica*	Spring Hollow	12
758	*P. kingii* subsp. *kaibabensis*	NF 758	20
AZT	*P. kingii* subsp. *kaibabensis*	AZ Trail	20
CIP	*P. kingii* subsp. *kaibabensis*	Culvert Pines	20
CrL	*P. kingii* subsp. *kaibabensis*	Crane Lake	20
DPt	*P. kingii* subsp. *kaibabensis*	Dog Point	20
DrL	*P. kingii* subsp. *kaibabensis*	Deer Lake	20
Hen	*P. kingii* subsp. *kaibabensis*	Henningsen	20
KbL	*P. kingii* subsp. *kaibabensis*	Kaibab Lodge	20
MV	*P. kingii* subsp. *kaibabensis*	Marble Viewpoint	20
Pvy	*P. kingii* subsp. *kaibabensis*	Pleasant Valley	20
TL	*P. kingii* subsp. *kaibabensis*	Three Lakes	20
VTL	*P. kingii* subsp. *kaibabensis*	VT Lake	20
429	*P. kingii* subsp. *latifolia*	NF429	20
DPk	*P. kingii* subsp. *latifolia*	Dry Park	20
Lk3	*P. kingii* subsp. *latifolia*	Leka3	20
LP	*P. kingii* subsp. *latifolia*	Locust Point	20
SHO	*P. kingii* subsp. *latifolia*	Snipe Hollow	13
MOC	*P. kingii* subsp. *latifolia*	Mammoth Creek	11
PgL	*P. kingii* subsp. *latifolia*	Panguitch Lake	10
LCY	*P. kingii* subsp. *latifolia*	Lee Canyon	15

### 2.2 Microsatellite Genotyping and Analysis

Thirteen microsatellite loci were used for genotyping, three developed for *Physaria fendleri* (A. Gray) O'Kane & Al‐Shehbaz (formerly *Lesquerella fendleri* (A. Gray) S. Watson; Salywon and Dierig 2006), five developed for *Physaria filiformis* (Rollins) O'Kane & Al‐Shehbaz (Edwards et al. 2021), and five developed for *Physaria obcordata* (Rollins) and *P. congesta* (Rollins) O'Kane & Al‐Shehbaz (formerly *Lesquerella congesta* Rollins) by one of the co‐authors (JRMN) using unpublished sequences shared by A. Salywon from work on *P. fendleri* (Table 2). An M13‐tag was added to the 5′ end of each forward primer for fluorescent labeling (Schuelke 2000). The PCR reaction consisted of 10 μL reaction volume with the following components: 0.2 mM dNTPs, 1x Promega PCR buffer, 1.5 mM to 3 mM MgCl_2_, 0.2 μM of M13 dye‐labeled primer and reverse primer, 0.02 μM of M13‐tagged forward primer, 0.5 U of Promega taq polymerase, and 20 ng of template DNA. The PCR reaction was performed with an initial denature temperature at 95°C for 5 min, followed by 35 cycles of 94°C denaturation for 30 s, 50°C–55°C annealing (see Table 2) for 30 s, 72°C extension for 30 s, and a final extension of 72°C for 4 min. Ten percent of the samples were randomly selected and amplified again to serve as replicates for detecting genotyping errors (Meirmans 2015). PCR products were genotyped using an ABI 3730 DNA Analyzer at the Field Museum, Chicago. Genotypes were scored using Microsatellite Analysis software (MSA) on the Thermo Fisher cloud based Connect Data Analysis platform (Thermo Fisher Scientific).

**TABLE 2:** Microsatellite primer used in this study. Primer sequences, repeat motif, fragment size, and PCR conditions are listed. Primers from Neale were developed from *Physaria obcordata* (Rollins) and *P. congesta* (Rollins) O'Kane & Al‐Shehbaz, those from Salywon and Dierig (2006) were developed for *P. fendleri* (A. Gray) O'Kane & Al‐Shehbaz, and those from Edwards et al. (2021) were developed for *P. filiformis* (Rollins) O'Kane & Al‐Shehbaz.

Locus	Repeat motif	Primer sequences	Allele size range	Annealing temperature (°C)	MgCl_2_ (mM)	Reference
LF6	(AAC)6	F: CCAAGCGCTCGTCGGAGAT	366–384	55	3.0	Neale (unpublished)
		R: GAACCGCGTTTTGCTGTTG				
LF14	(AG)9	F: GACCGTACATCGGAAACTGC	204–226	50	3.0	Salywon and Dierig (2006)
		R: CGTATGGTGAATTTCAAGAC				
LF20	(TATG5	F: TTCCTTCCTTACTCACATCC	152–186	50	3.0	Salywon and Dierig (2006)
		R: TGAAGGTCCTAAGCATATC				
LF24	(TG)12	F: TGTAATGGGATGACATGTGG	257–291	55	3.0	Salywon and Dierig (2006)
		R: CTTGCGATGAAAAGAGCTGC				
LF25	(GA)12(GT) (GA)2	F: TTGAGAGCATCGCTGCAATC	300–330	55	1.5	Neale (unpublished)
		R: CGTAATCTCTTCATCGGACG				
LF27	(GAA)9	F: CATCATCTAGAGATGAGGAG	230–240	55	3.0	Neale (unpublished)
		R: GAATATGGGTTTACAAGTGG				
LF29	(TG)12	F: TTGCAGTTGCATACCGTTGC	240–250	55	3.0	Neale (unpublished)
		R: ATGTGATCCATGTGTCTGCC				
LF35	(TTTG)2 (TG)7	F: CGATCGATCAGAATTGAAGGC	340–350	55	3.0	Neale (unpublished)
		R: GTCTTAGTGGGTATTCACTC				
PF8	(AG)10	F: ACCAATTGCAGATGAAGGCG	183–194	55	3.0	Edwards et al. (2021)
		R: AGTTCTTTGTTTCGAGGGTCC				
PF33	(AG9	F: CCAGATTCCGACACGACTTG	113–131	55	3.0	Edwards et al. (2021)
		R: AACCCACATCCCACGCATC				
PF37	(AG)9	F: GCTTCGTATTCCTGATGCCG	147–160	55	3.0	Edwards et al. (2021)
		R: CGTTTACCAAATCACTGCGC				
PF62	(AG8	F: ACCTTCTTCGTCATCTCCGG	136–142	55	3.0	Edwards et al. (2021)
		R: CATCTCCGCACCTTTACACC				
PF82	(AG)14	F: TCCTCCACCGTCAAGTCATC	141–156	55	3.0	Edwards et al. (2021)
		R: CACACACCATCTTCTTTGCAC				

Standard population genetics statistics such as the average number of alleles, effective number of alleles, observed heterozygosity, expected heterozygosity, inbreeding coefficients, and private alleles were estimated using GenAlEx v.6.5 (Peakall and Smouse 2012) and R package PopGenReport (Adamack and Gruber 2014). Linkage disequilibrium was tested via GENEPOP v.4.8.3 to detect potential non‐random association of alleles between pairs of loci in each population (Rousset 2008). Hardy–Weinberg equilibrium was examined at each locus in each population using Fisher's exact tests in GENEPOP. Pairwise genetic differentiation among populations was estimated with *F*_ST_ and *G'*_ST_ (10,000 permutations) using GenoDive v3.0 (Meirmans 2020). Principle component analysis (PCoA) was performed in GenAlEx to visualize genetic similarities among individuals of different *Physaria* taxa.

The Bayesian clustering analysis software STRUCTURE v.2.3.4 was used to identify genetic clusters based on multilocus genotypes without a priori assignment of individual plants to populations or taxonomic groups (Pritchard, Stephens, and Donnelly 2000). After a burn‐in of 500 k runs, 1000 k MCMC replicates were performed with the admixture ancestry model and correlated allele frequency model. Structure analyses were conducted without using sampling location to inform priors. The number of genetic clusters (*K*) was tested from *K* = 1 to 20 with 10 iterations per *K*. We used STRUCTURE SELECTOR (Li and Liu 2018) to select best *K* using the estimates DeltaK (Evanno, Regnaut, and Goudet 2005) and MaxMedK (Puechmaille 2016). STRUCTURE bar plots were constructed using CLUMPAK for visualizing genetic structure (Kopelman et al. 2015). We conducted three sets of STRUCTURE analyses. First, we included all *Physaria* samples (*P. arizonica* and *P. kingii*) to detect differentiation between the two *Physaria* species. Second, we conducted an analysis that only included *P. kingii* samples to examine intraspecific genetic structure and subspecific delimitations. Finally, STRUCTURE analysis was conducted on *P. arizonica* samples (*K* = 1 to 10) to investigate genetic structure within this species.

Analysis of molecular variance (AMOVA) was conducted to examine genetic differentiation and hierarchical structure within and among *Physaria* populations. Three AMOVA analyses were conducted using GenAlEx with these groupings: (1) Samples grouped by species to detect genetic differentiation between *P. kingii* (369 samples from 20 sites) and *P. arizonica* (94 samples from six sites); (2) *P. kingii* samples grouped into two subspecies *P. kingii* subsp. *kaibabensis* (240 samples from 12 sites) and *P. kingii* subsp. *latifolia* (129 samples from eight sites) to examine hierarchical population structure within *P. kingii*; (3) *P. kingii* samples collected only from the Kaibab Plateau (333 samples from 17 sites) grouped into two subspecies *P. kingii* subsp. *kaibabensis* (240 samples from 12 sites) and *P. kingii* subsp. *latifolia* (93 samples from 5 sites). Intraspecific population structure of *P. arizonica* samples was not examined with AMOVA due to the limited number of collection sites.

For *P. kingii*, isolation by distance (IBD) was examined to determine whether spatially distant populations are more genetically differentiated (Slatkin 1987, 1993). We calculated distances between sampling sites from unprojected geographic coordinates using the geosphere package (Hijmans, Williams, and Vennes 2019) in R version 4.3.2 (R Core Team 2023). A Mantel test was conducted in GenAlEx to see if genetic distances (estimated as *F*_ST_) were correlated with the geographical distances among (a) All *P. kingii* populations and (b) *P. kingii* only from the Kaibab Plateau. Due to the low number of sampled sites (*n* = 6), we did not conduct isolation by distance analysis for *P. arizonica*.

## 3 Results

Microsatellite diversity of 463 *Physaria* samples (94 *P. arizonica*, 240 *P. kingii* subsp. *kaibabensis*, and 129 *P. kingii* subsp. *latifolia*) averaged across 13 microsatellite loci is summarized in Table 3. Descriptive statistics for each locus by species are provided in Supplement 1 and for each sampling location in Supplement 2. The mean number of alleles per locus (A), effective number of alleles averaged across loci (A_e_), observed heterozygosity (H_O_), and expected heterozygosity (H_E_) were similar in the *P. arizonica*, *P. kingii* subsp. *kaibabensis*, and *P. kingii* subsp. *latifolia* populations. The mean number of alleles (A) and the mean number of effective alleles (A_e_) of each population ranged from 1.8 to 4.8 and 1.4 to 2.8, respectively. Observed heterozygosity (H_O_) ranged from 0.263 to 0.526, with mean H_O_ equal to 0.439. Expected heterozygosity (H_E_) ranged from 0.316 to 0.573, with mean H_E_ equal to 0.460. Linkage disequilibrium was not detected between the loci. Overall, populations did not show significant deviations from the Hardy–Weinberg equilibrium after applying the Bonferroni correction (*p* < 0.00015), except for populations DPt and DrL at locus LF35, and population PgL at locus LF14 (Supplement 2). The highest number of private alleles (13) was found in *P. kingii* subsp. *latifolia* at LCY, a site near Las Vegas, Nevada, about 300 km from the Kaibab Plateau. Pairwise genetic divergence (*F*_ST_) between sampled sites ranged between 0 and 0.68 (Supplement 3), with values among *P. arizonica* populations ranging between 0.05 and 0.42. Among *P. kingii* subsp. *kaibabensis* populations, pairwise *F*_ST_ values ranged between 0 and 0.16. The range for *P. kingii* subsp. *latifolia* sampled sites were 0.01 to 0.49, with lower values for samples from the Kaibab Plateau (between 0.01 and 0.21). This suggests limited gene flow between populations on the Kaibab Plateau and those outside the plateau (PgL, MOC, and LCY).

**TABLE 3:** Summary of microsatellite diversity statistics across 13 loci for 463 *Physaria* samples: The number of samples (*N*), mean number of alleles per locus (*A*), mean effective number of alleles per locus (*A*_*e*_), number of private alleles (*A*_*P*_), observed heterozygosity (*H*_*O*_), expected heterozygosity (*H*_*E*_), and fixation index (*F*).

Species	Sampling location	*N*	*A*	*A*_*e*_	*A*_*P*_	*H*_*O*_	*H*_*E*_	*F*
*P. arizonica*	462	20	3.154	2.010	3	0.461	0.507	0.009
	HR	20	4.385	2.731	7	0.490	0.539	0.077
	LeF	20	3.077	1.672	3	0.418	0.458	0.020
	LFR	10	2.538	1.880	3	0.420	0.521	0.163
	Nre	12	2.615	2.021	0	0.437	0.422	0.023
	SPH	12	1.846	1.423	4	0.263	0.316	0.096
*P. kingii* subsp. *kaibabensis*	758	20	4.000	2.746	1	0.441	0.498	0.185
	AZT	20	4.615	2.701	0	0.503	0.573	0.116
	CIP	20	4.308	2.768	0	0.511	0.520	0.016
	CrL	20	4.308	2.284	0	0.481	0.505	0.031
	DPt	20	4.154	2.584	0	0.496	0.538	0.066
	DrL	20	4.231	2.352	0	0.418	0.497	0.145
	Hen	20	4.231	2.545	1	0.500	0.547	0.106
	KbL	20	3.385	2.313	0	0.442	0.486	0.102
	MV	20	4.538	2.384	1	0.462	0.477	0.016
	Pvy	20	4.308	2.558	0	0.511	0.512	−0.009
	TL	20	4.538	2.408	1	0.437	0.496	0.087
	VTL	20	4.385	2.701	2	0.526	0.541	0.020
*P. kingii* subsp. *latifolia*	429	20	4.769	2.824	1	0.523	0.528	−0.006
	DPk	20	4.692	2.706	1	0.525	0.546	0.016
	Lk3	20	4.538	2.694	1	0.521	0.557	0.070
	LP	20	2.462	1.744	2	0.353	0.419	0.010
	SHO	13	4.308	2.665	2	0.512	0.512	−0.012
	MOC	11	3.231	2.081	4	0.366	0.490	0.289
	PgL	10	3.385	2.404	1	0.444	0.557	0.183
	LCY	15	3.385	2.019	13	0.346	0.419	0.208

**FIGURE 3:**
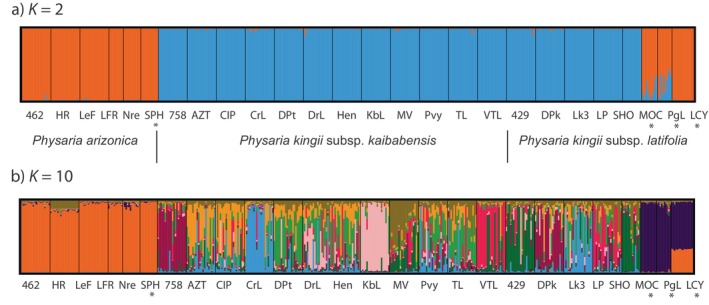
Bar plot results from STRUCTURE for all *Physaria* samples (*n* = 463). Each column represents the genetic assignment of a single individual. We present the results from the optimal models using best *K* = 2 (DeltaK, Evanno, Regnaut, and Goudet 2005) and *K* = 10 (MaxMedK, Puechmaille 2016). Sampling sites marked by * (SPH, MOC, PgL, and LCY) were located outside of the Kaibab Plateau.

In our STRUCTURE analysis, for all *Physaria* samples combined, the DeltaK method (Evanno, Regnaut, and Goudet 2005) indicated that the optimal number of clusters was *K* = 2 (Supplement 4a) and the MaxMedK (Puechmaille 2016) method indicated *K* = 10 (Supplement 4b). For *K* = 2, one cluster comprised *P. arizonica* (shown in orange in Figure 3a), and the other cluster comprised both *P. kingii* subsp. *kaibabensis* and *P. kingii* subsp. *latifolia* from sites on the Kaibab Plateau (shown in blue in Figure 3a). *P. kingii* subsp. *latifolia* from outside the Kaibab Plateau were either assigned ancestry with *P. arizonica* (LCY) or showed mixed ancestry (MOC and PgL). For *K* = 10, *P. arizonica* again formed a single cluster, but the *P. kingii* subsp. *kaibabensis* and *P. kingii* subsp. *latifolia* from sites on the Kaibab Plateau showed mixed ancestry in several clusters (Figure 3b). *P. kingii* subsp. *latifolia* from outside the Kaibab Plateau were either assigned to a separate cluster (MOC and PgL) or had mixed ancestry with *P. arizonica* (LCY). Setting the number of clusters to *K* = 3 (Supplement 4c) and *K* = 4 (Supplement 4d) provides further support for a single *P. kingii* taxon on the plateau that is distinct from *P. arizonica* and *P. kingii* subsp. *latifolia* off the plateau.

**FIGURE 4:**
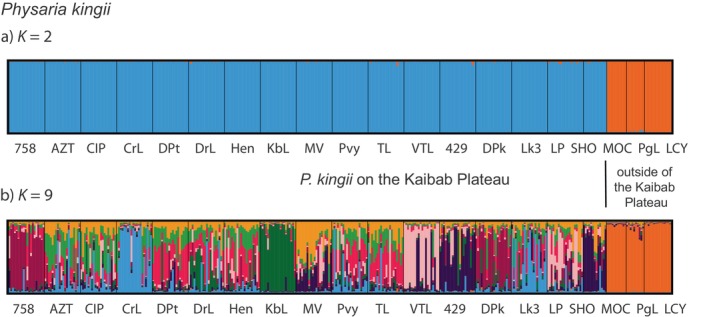
Bar plot results from STRUCTURE for *Physaria kingii* samples (*n* = 369). Each column represents the genetic assignment of a single individual. We present the results from the optimal models using best *K* = 2 (DeltaK, Evanno, Regnaut, and Goudet 2005) and *K* = 9 (MaxMedK, Puechmaille 2016).

For STRUCTURE analysis on *P. kingii* alone, the DeltaK method indicated that the optimal number of clusters was *K* = 2 (Supplement 5a) and the MaxMedK method indicated *K* = 9 (Supplement 5b). For both *K* = 2 and *K* = 9 (Figure 4a,b), *P. kingii* populations outside of the Kaibab Plateau (MOC, PgL, and LCY) formed a distinct genetic cluster from the *P. kingii* populations on the Kaibab Plateau. For *K* = 9 (Figure 4b), some clustering was seen among the Kaibab Plateau samples, but clusters did not correspond to subspecies designation; genetic differentiation was not evident between *P. kingii* subsp.

**FIGURE 5:**
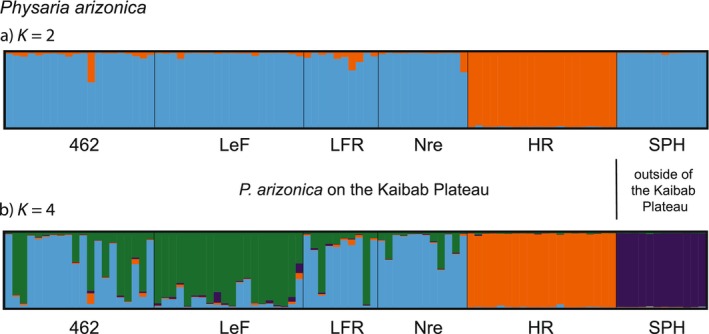
Bar plot results from STRUCTURE for *Physaria arizonica* (*n* = 94). Each column represents the genetic assignment of a single individual. We present the results from the optimal models using best *K* = 2 (DeltaK, Evanno, Regnaut, and Goudet 2005) and *K* = 4 (MaxMedK, Puechmaille 2016).

For STRUCTURE analysis of *P. arizonica*, the Delta *K* method indicated best *K* = 2 (Supplement 6a) and MaxMedK indicated best *K* = 4 (Supplement 6b). The resulting bar plots reflect the geographic separation of the *P. arizonica* populations (Figure 5a,b). On the Kaibab Plateau, genetic admixture was evident among 462, LeF, LFR, and Nre populations. The HR population on the Kaibab Plateau and the SPH population outside of the Kaibab Plateau each formed a distinct cluster.

**FIGURE 6:**
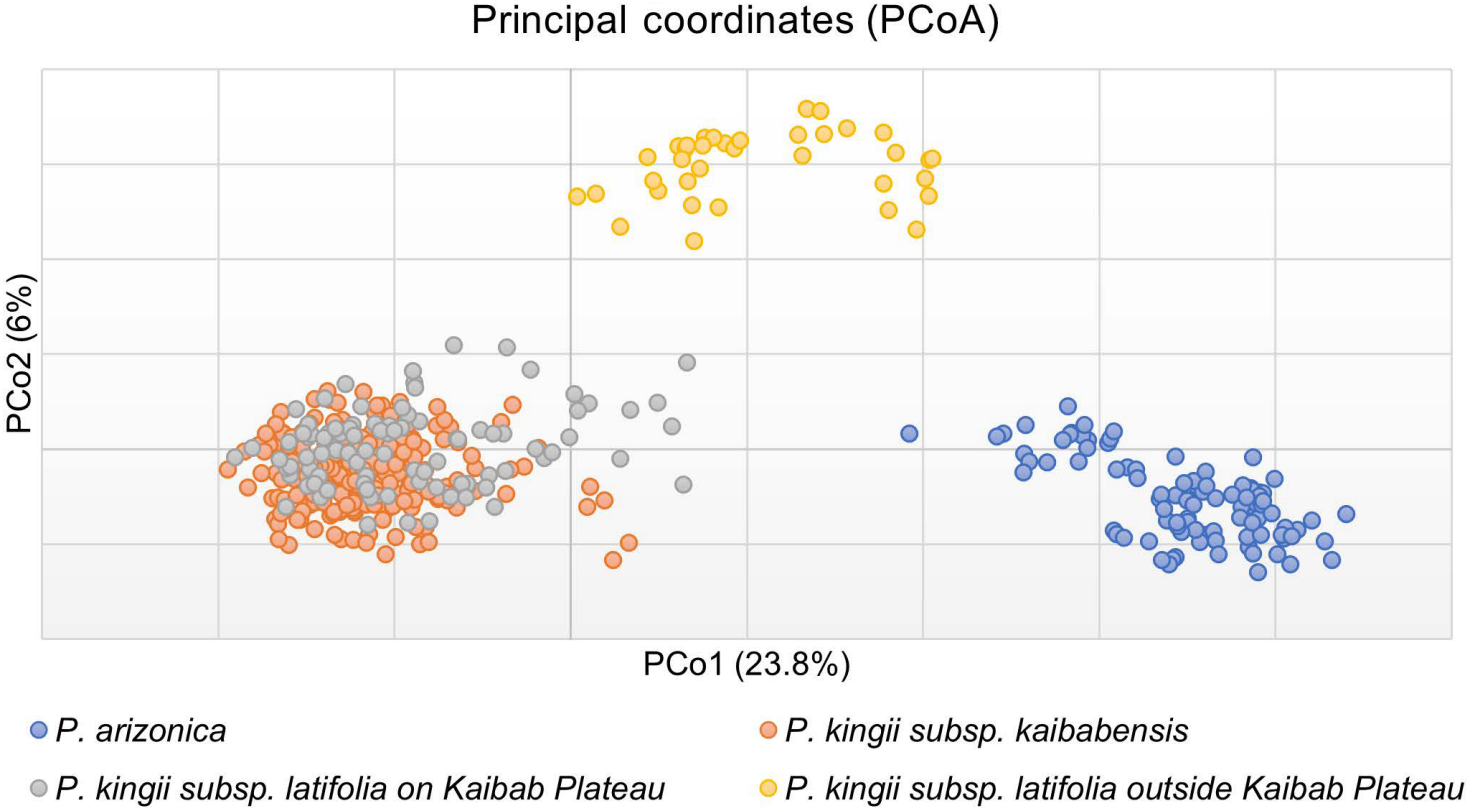
Principal coordinates analysis (PCoA) for all *Physaria* individuals. PCo1 and PCo2 account for 23.8% and 6.0% of genetic variation, respectively.

Results from AMOVA when including all *Physaria* samples indicated that 22% of the genetic variation was partitioned between species (Table 4). Only 4% of the variance was partitioned between subspecies of *P. kingii*, dropping to 1% when only sites on the Kaibab Plateau were included. Principal coordinates analysis (PCoA) further supports the STRUCTURE and AMOVA analyses (Figure 6). PCoA axes 1 and 2 account for 23.8% and 6.0% of genetic variation, respectively. *P. arizonica* samples cluster together. There were two groups of *P. kingii*, one comprised of *P. kingii* subsp. *latifolia* from outside the Kaibab Plateau, and one comprised of both subspecies from sites on the plateau.

**TABLE 4:** Summary of AMOVA analysis among *Physaria arizonica*, *P. kingii* subsp. *kaibabensis,* and *P. kingii* subsp. *latifolia* populations. The analyses were conducted in three ways, as described in the text.

Population comparison	Source of variation	Degrees of freedom	Sum of squares	Estimated variance	% of variance	Fixation indices
(1) *P. arizonica* & *P. kingii*	Among species	2	717.26	1.15	22	*F*_RT_ = 0.22
	Among populations within species	23	723.71	0.78	15	*F*_SR_ = 0.20
	Within populations	437	1619.35	0.47	9	*F*_ST_ = 0.37
(2) *P. kingii* subsp. *kaibabensis* & *P. kingii* subsp. *latifolia*	Among subspecies	1	82.05	0.15	4	*F*_RT_ = 0.04
	Among populations within subspecies	18	548.17	0.73	17	*F*_SR_ = 0.18
	Within populations	349	1289.77	0.37	9	*F*_ST_ = 0.21
(3) *P. kingii* subsp. *kaibabensis* & *P. kingii* subsp. *latifolia* within the Kaibab Plateau	Among subspecies	1	29.50	0.04	1	*F*_RT_ = 0.01
	Among populations within subspecies	15	292.01	0.40	11	*F*_SR_ = 0.11
	Within populations	316	1164.06	0.32	8	*F*_ST_ = 0.12

**FIGURE 7:**
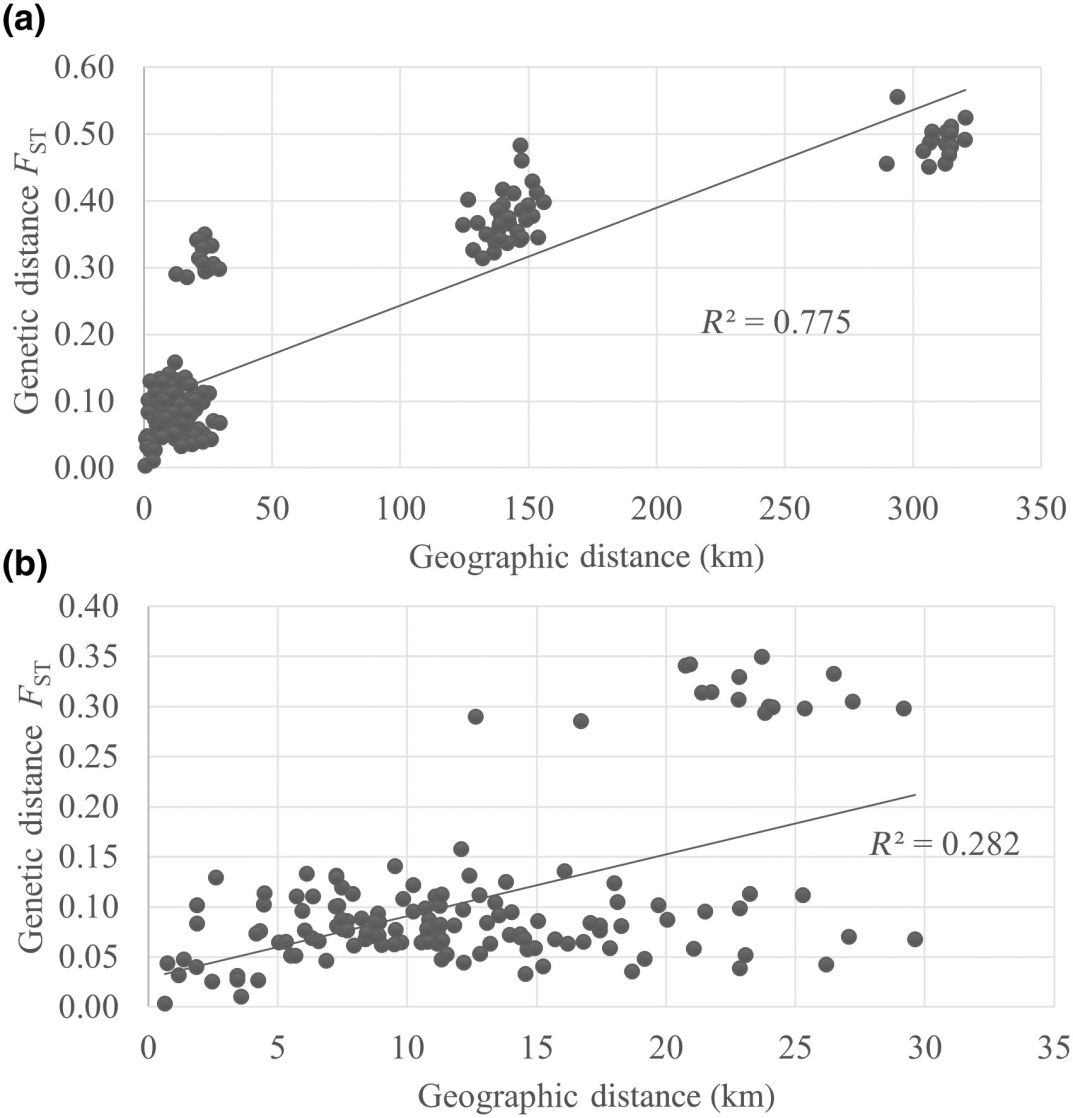
Isolation by distance for: (a) All *Physaria kingii* populations and (b) *P. kingii* populations on the Kaibab Plateau. The pairwise genetic distances, as measured by *F*_ST_, is plotted against the pairwise geographical distances (in kilometers) among *P. kingii* sampling sites. Each point represents a pairwise comparison between population pairs. (a) All *P. kingii* populations (*n* = 20). (b) *Physaria kingii* populations on the Kaibab Plateau (*n* = 17).

For *P. kingii*, genetic distance *F*_ST_ was correlated with geographical distance (*R* ^2^ = 0.78, Mantel test, *p* < 0.001; Figure 7a). This correlation was significantly reduced after the three *P. kingii* subsp. *latifolia* populations outside of the Kaibab Plateau (MOC, PgL, LCY) were removed from the IBD analysis, but the relationship was still statistically supported (*R* ^2^ = 0.28, Mantel test, *p* = 0.017; Figure 7b).

## 4 Discussion

We used nuclear microsatellite markers to investigate the genetic diversity and population structure of three *Physaria* taxa distributed on and off the Kaibab Plateau in Arizona. Previous investigations had reported that two subspecies of *P. kingii*, *P. kingii*. subsp. *latifolia* and *P. kingii*. subsp. *kaibabensis*, co‐occur on the Kaibab Plateau. Additionally, gene flow was hypothesized to have occurred with another *Physaria* species on the plateau, *P. arizonica*. *P. kingii* subsp. *kaibabensis* is of conservation concern because its range is restricted to the plateau and it is threatened by habitat destruction and other disturbances (Molano‐Flores and Coons 2020). We sampled *P. kingii* at 17 sites on the Kaibab Plateau, and plants from five of these collection sites were morphologically assigned to *P. kingii* subsp. *latifolia* according to Minnaert‐Grote (2014). The remaining plants from 12 sites were assigned to *P. kingii* subsp. *kaibabensis*. Despite these subspecies designations, we found that there is currently a single genetic cluster of *P. kingii* on the Kaibab Plateau, and it is distinct from *P. kingii* subsp. *latifolia* sites sampled off the plateau (Figures 3 and 4). Although some genetic structure emerged at higher *K* values, it did not correspond to subspecies designations, and most individuals showed mixed ancestry. The AMOVA results (Table 4) and the PCoA plot (Figure 6) provide additional support for a single genetic group of *P. kingii* on the plateau.

Our results call into question the current taxonomic designations of two *P. kingii* subspecies on the Kaibab Plateau and suggest that the morphological character primarily used to delineate the taxa, flower color, is unreliable. *P. kingii* subsp. *kaibabensis* has cream‐white or white flowers while *P. kingii* subsp. *latifolia* has yellow flowers. As mentioned above, however, *P. kingii* subsp. *latifolia* on the Kaibab Plateau produce flower colors ranging from yellow to white (O'Kane 2010). A study of *P. fendleri* identified a cream‐colored flower mutation controlled by a single allele that was recessive to wild‐type yellow flowers (Dierig, Salywon, and de Rodriquez 2006). If cream and white flowers in *P. kingii* are similarly under the control of a single gene, it is likely a polymorphic trait that may simply differ in frequency among populations. Besides flower color, there are additional morphological characters in which *P. kingii* subsp. *latifolia* plants on the plateau are more similar to *P. kingii* subsp. *kaibabensis* than to *P. kingii* subsp. *latifolia* plants off the plateau, including the vestiture of the inner valves (glabrous, rather than pubescent), a greater number of ovules per ovary, and a shorter style (Minnaert‐Grote 2014; O'Kane 2010). While we did not measure these traits on the specimens we collected for genotyping (and not all plants had reproductive structures at the time of sampling), additional research should assess the efficacy of these traits for distinguishing *P. kingii* subspecies.

Field observations during the blooming season suggest that pollinator preferences do not limit gene flow between the populations designated as *P. kingii* subsp. *kaibabensis* and *P. kingii* subsp. *latifolia* on the plateau (Molano‐Flores and Coons 2020). Pollen is dispersed primarily by bees, and the two *P. kingii* subspecies produce similar visual cues to attract pollinators despite slight differences in flower coloration. In contrast, no recent gene flow seems to be occurring between *P. kingii* and *P. arizonica* on the plateau, as evidenced by clear genetic differentiation of the two species (Figures 3 and 6). *P. arizonica* occurs on the north side of the Kaibab Plateau, while the *P. kingii* collection sites were distributed on the south side (Figure 2), so spatial separation may play a role in limiting gene dispersal between the species. However, a recent investigation of pollen morphology reports that the equatorial diameter of *P. arizonica* pollen was significantly wider than *P. kingii* (Wallace, Coons, and Molano‐Flores 2021) and suggests that gametic prezygotic isolation likely limits cross‐pollination between *P. arizonica* and *P. kingii*.

Our results do not support a hybrid origin for *P. kingii* subsp. *latifolia* on the Kaibab plateau, as suggested by Minnaert‐Grote (2014). However, we cannot rule out a hybridization event (and subsequent introgression) in the distant past. It is difficult to reconcile our results with the recent report of a tetraploid *P. kingii* subsp. *latifolia* plant collected from the edge of the Kaibab Plateau (Salywon, Rebman, and Dierig 2022). (For spatial reference, the tetraploid *P. kingii* subsp. *latifolia* plant was sampled near our *P. arizonica* collection site Nre). While we did not assess chromosome counts of any of our samples, we did not find more than two alleles in any of the microsatellite genotypes of *P. kingii* subsp. *latifolia* which could indicate polyploidy. The genetic similarity of *P. kingii* subsp. *kaibabensis* and *P. kingii* subsp. *latifolia* on the plateau also argues against genetic isolation based on differences in ploidy. However, further work on chromosome counts of *P. kingii* subsp. *latifolia* on or near the Kaibab Plateau should be undertaken.

Samples designated as *P. kingii* subsp. *latifolia* on the Kaibab Plateau were genetically differentiated from *P. kingii* subsp. *latifolia* samples collected off the plateau. Although our sampling off the plateau was limited to three sites, the distinction is at least partially supported by DNA sequencing and morphology (Minnaert‐Grote 2014). Genetic differentiation and gene flow limitations for Kaibab Plateau plant populations have been reported for several taxa (reviewed in Rink, Hodgson, and Phillips 2020). Our findings for the other species in our study, *P. arizonica*, further indicate that the Kaibab Plateau creates a barrier to gene flow. Populations on the plateau were more similar to one another than those off of the plateau (Figure 5). The comparison of collections from sites Nre and HR is interesting. The collection sites were the nearest pair of *P. arizonica* populations (Figure 2), separated by approximately 5 km, but they occur near the transition from the Kaibab Plateau to the desert floor; Nre is at a greater elevation on the plateau, and HR is nearer to the desert floor. The two populations are clearly genetically differentiated (Figure 5), with Nre belonging to the same genetic cluster as a population 94 km to the north (SPH).

We acknowledge that the findings of our research should be interpreted with caution due to several limitations. Genetic variation in *P. kingii* subsp. *kaibabensis* and *P. kingii* subsp. *latifolia* is not fully represented by the microsatellite markers we used in this study. Microsatellites are neutral markers that do not detect adaptive differentiation in population due to local selection, but rather reflect processes related to gene flow and genetic drift. Genomic studies could provide a better understanding of genetic divergence and adaptive evolution in *Physaria*. *P. kingii* subsp. *latifolia* is widespread and common in western North America. More *P. kingi* subsp. *latifolia* should be sampled from outside of the Kaibab Plateau to better understand genetic structure and morphological diversity throughout its range, especially from other plateaus to the north that are part of the same chain.

Despite these caveats, we found substantial evidence for a distinct evolutionary lineage of *P. kingii* on the Kaibab Plateau. Our results show that *P. kingii* populations on the Kaibab Plateau are genetically diverse and genetically well‐differentiated from a co‐occurring congener and from other *P. kingii* populations. We recommend that the subspecies designation of *P. kingii* subsp. *kaibabensis* be revised to include all the *P. kingii* populations on the Kaibab Plateau and that it to be considered a taxonomic unit that warrants legal protection. Even though uncertainties in the taxonomic identification and subspecies delineations among the *P. kingii* subspecies remain, we feel it is imperative to continue developing conservation management planning for *P. kingii* subsp. *kaibabensis*. This subspecies has a restricted range and is threatened by habitat destruction, climate change, and nonnative species (Rink, Hodgson, and Phillips 2020). Our findings that it is genetically unique provides additional support for its protection.

## Author Contributions

**Jer Pin Chong:** conceptualization (lead), data curation (lead), formal analysis (lead), investigation (lead), methodology (lead), validation (lead), visualization (lead), writing – original draft (lead). **Jamie Minnaert‐Grote:** validation (supporting), writing – review and editing (supporting). **David N. Zaya:** conceptualization (supporting), data curation (supporting), formal analysis (supporting), methodology (supporting), validation (supporting), visualization (supporting), writing – original draft (supporting). **Mary V. Ashley:** conceptualization (supporting), data curation (supporting), formal analysis (supporting), investigation (supporting), methodology (supporting), validation (supporting), visualization (supporting), writing – review and editing (supporting). **Janice Coons:** funding acquisition (supporting), investigation (supporting), methodology (supporting), project administration (supporting), writing – review and editing (supporting). **Jennifer M. Ramp Neale:** methodology (supporting), writing – review and editing (supporting). **Brenda Molano‐Flores:** conceptualization (supporting), funding acquisition (lead), investigation (equal), methodology (supporting), project administration (lead), writing – review and editing (supporting).

## Conflicts of Interest

The authors declare no conflicts of interest.

## Supporting information


Data S1

## Data Availability

Genotype and location data used as the basis of all analyses presented in this study are available through the Illinois Data Bank: https://doi.org/10.13012/B2IDB‐2540221_V1. Genotype data include microsatellite allele size for all loci. For locality data (geographic coordinates) and other information for each sampling location contact the corresponding author. Detailed metadata are also included with this submission.

## References

[ece370523-bib-0001] Adamack, A. T. , and B. Gruber . 2014. “PopGenReport: Simplifying Basic Population Genetic Analyses in R.” Methods in Ecology and Evolution 5, no. 4: 384–387. 10.1111/2041-210X.12158.

[ece370523-bib-0002] Al‐Shehbaz, I. A. 2012. “A Generic and Tribal Synopsis of the Brassicaceae (Cruciferae).” Taxon 61, no. 5: 931–954. 10.1002/tax.615002.

[ece370523-bib-0003] Al‐Shehbaz, I. A. , and S. L. O'Kane . 2002. “ *Lesquerella* is United With *Physaria* (Brassicaceae).” Novon 12, no. 3: 319‐329. 10.2307/3393073.

[ece370523-bib-0048] Arizona Game and Fish Department . 2024. “Special Status Species. Heritage Data Management System.” Updated 30 July 2024. https://www.azgfd.com/wildlife‐conservation/planning‐for‐wildlife/planning‐for‐wildlife‐wildlife‐friendly‐guidelines/planning‐for‐wildlife‐species‐lists/.

[ece370523-bib-0005] Backs, J. R. , M. Terry , and M. V. Ashley . 2016. “Using Genetic Analysis to Evaluate Hybridization as a Conservation Concern for the Threatened Species *Quercus hinckleyi* C.H. Muller (Fagaceae).” International Journal of Plant Sciences 177, no. 2: 122–131. 10.1086/684177.

[ece370523-bib-0006] Bradbury, D. , R. M. Binks , A. Webb , and M. Byrne . 2023. “Defining Conservation Units in a Species Complex With Genomic‐Taxonomic Discordance: A Case Study of *Conospermum caeruleum* (Proteaceae).” Biodiversity and Conservation 32, no. 6: 1949–1975. 10.1007/s10531-023-02585-z.

[ece370523-bib-0007] DeMauro, M. M. 1993. “Relationship of Breeding System to Rarity in the Lakeside Daisy (*Hymenoxys acaulis var. glabra*).” Conservation Biology 7, no. 3: 542–550. 10.1046/j.1523-1739.1993.07030542.x.

[ece370523-bib-0008] Dierig, D. A. , A. M. Salywon , and D. J. de Rodriquez . 2006. “Registration of a Mutant *Lesquerella* Genetic Stock With Cream Flower Color.” Crop Science 46, no. 4: 1836–1837. 10.2135/cropsci2006.02-0105.

[ece370523-bib-0009] Edwards, C. E. , B. C. Tessier , J. F. Swift , et al. 2021. “Conservation Genetics of the Threatened Plant Species *Physaria filiformis* (Missouri Bladderpod) Reveals Strong Genetic Structure and a Possible Cryptic Species.” PLoS One 16: e0247586. 10.1371/journal.pone.0247586. 33705416 PMC7951829

[ece370523-bib-0010] Evanno, G. , S. Regnaut , and J. Goudet . 2005. “Detecting the Number of Clusters of Individuals Using the Software STRUCTURE: A Simulation Study.” Molecular Ecology 14, no. 8: 2611–2620. 10.1111/j.1365-294X.2005.02553.x. 15969739

[ece370523-bib-0011] Finger, A. , C. J. Kettle , C. N. Kaiser‐Bunbury , et al. 2011. “Back From the Brink: Potential for Genetic Rescue in a Critically Endangered Tree.” Molecular Ecology 20, no. 18: 3773–3784. 10.1111/j.1365-294X.2011.05228.x. 21883581

[ece370523-bib-0012] Hanson, J. O. , R. A. Fuller , and J. R. Rhodes . 2019. “Conventional Methods for Enhancing Connectivity in Conservation Planning Do Not Always Maintain Gene Flow.” Journal of Applied Ecology 56, no. 4: 913–922. 10.1111/1365-2664.13315.

[ece370523-bib-0013] Harley, R. M. , R. C. Rollins , and E. A. Shaw . 1974. “The Genus *Lesquerella* (Cruciferae) in North America.” Kew Bulletin 29, no. 3: 625. 10.2307/4108018.

[ece370523-bib-0014] Hijmans, R. J. , E. Williams , and C. Vennes . 2019. “Geosphere: Spherical Trigonometry.” R package version 1.5–10. In package geosphere.

[ece370523-bib-0015] Holmgren, N. H. 2004. “Lectotypifications and New Combinations in North American Brassicaceae.” Brittonia 56, no. 3: 245–248. 10.1663/0007-196X(2004)056[0245:LANCIN]2.0.CO;2.

[ece370523-bib-0016] Kelly, E. , and B. L. Phillips . 2016. “Targeted Gene Flow for Conservation.” Conservation Biology 30, no. 2: 259–267. 10.1111/cobi.12623. 26332195

[ece370523-bib-0017] Kim, E. S. , D. N. Zaya , J. B. Fant , and M. V. Ashley . 2015. “Genetic Factors Accelerate Demographic Decline in Rare *Asclepias* Species.” Conservation Genetics 16, no. 2: 359–369. 10.1007/s10592-014-0663-3.

[ece370523-bib-0018] Kopelman, N. M. , J. Mayzel , M. Jakobsson , N. A. Rosenberg , and I. Mayrose . 2015. “CLUMPAK: A Program for Identifying Clustering Modes and Packaging Population Structure Inferences Across K.” Molecular Ecology Resources 15, no. 5: 1179–1191. 10.1111/1755-0998.12387. 25684545 PMC4534335

[ece370523-bib-0019] Li, Y. L. , and J. X. Liu . 2018. “StructureSelector: A Web‐Based Software to Select and Visualize the Optimal Number of Clusters Using Multiple Methods.” Molecular Ecology Resources 18, no. 1: 176–177. 10.1111/1755-0998.12719. 28921901

[ece370523-bib-0020] Meirmans, P. G. 2015. “Seven Common Mistakes in Population Genetics and How to Avoid Them.” Molecular Ecology 24, no. 13: 3223–3231. 10.1111/mec.13243. 25974103

[ece370523-bib-0021] Meirmans, P. G. 2020. “GENODIVE Version 3.0: Easy‐To‐Use Software for the Analysis of Genetic Data of Diploids and Polyploids.” Molecular Ecology Resources 20, no. 4: 1126–1131. 10.1111/1755-0998.13145. 32061017 PMC7496249

[ece370523-bib-0022] Minnaert‐Grote, J. 2014. “The Phylogenetics and Systematics of *Physaria kingii* (Brassicaceae).” MS thesis, University of Northern Iowa, Cedar Falls.

[ece370523-bib-0023] Molano‐Flores, B. , and J. Coons . 2020. “Reproductive Ecology of *Physaria kingii* Subsp. *kaibabensis*, an Endemic Species of the Kaibab Plateau, USA.” Natural Areas Journal 40, no. 4: 345–354. 10.3375/043.040.0407.

[ece370523-bib-0049] NatureServe . 2024. “NatureServe Network Biodiversity Location Data accessed through NatureServe Explorer [web application].” Arlington, Virginia: NatureServe. https://explorer.natureserve.org/. (Accessed: Nov 9, 2024).

[ece370523-bib-0025] O'Kane, S. L. 2007. “ *Physaria scrotiformis* (Brassicaceae), a New High‐Elevation Species From Southwestern Colorado and New Combinations in *Physaria* .” Novon 17, no. 3: 376–382. 10.3417/1055-3177(2007)17[376:PSBANH]2.0.CO;2.

[ece370523-bib-0026] O'Kane, S. L. 2010. “Physaria.” In Flora of North America North of Mexico, 16+ vols, Vol. 7, edited by Flora of North Editorial Committee . 1993+, 616–665. New York and Oxford.

[ece370523-bib-0027] Peakall, R. , and P. E. Smouse . 2012. “GenALEx 6.5: Genetic Analysis in Excel. Population Genetic Software for Teaching and Research‐An Update.” Bioinformatics 28, no. 19: 2537–2539. 10.1093/bioinformatics/bts460. 22820204 PMC3463245

[ece370523-bib-0028] Pritchard, J. K. , M. Stephens , and P. Donnelly . 2000. “Inference of Population Structure Using Multilocus Genotype Data.” Genetics 155, no. 2: 945–959. 10.1093/genetics/155.2.945. 10835412 PMC1461096

[ece370523-bib-0029] Puechmaille, S. J. 2016. “The Program Structure Does Not Reliably Recover the Correct Population Structure When Sampling is Uneven: Subsampling and New Estimators Alleviate the Problem.” Molecular Ecology Resources 16, no. 3: 608–627. 10.1111/1755-0998.12512. 26856252

[ece370523-bib-0030] R Core Team . 2023. R: A Language and Environment for Statistical Computing. https://www.R‐project.org/. Vienna, Austria: R Foundation for Statistical Computing.

[ece370523-bib-0031] Rasmussen, D. I. 1941. “Biotic Communities of Kaibab Plateau, Arizona.” Ecological Monographs 11, no. 3: 229–275. 10.2307/1943204.

[ece370523-bib-0032] Rink, G. R. , W. Hodgson , and B. G. Phillips . 2020. “Checklist of the Vascular Flora of the Kaibab Plateau, Coconino County, Arizona.” Monographs of the Western North American Naturalist 12, no. 1: 1–52. 10.3398/042.012.0101.

[ece370523-bib-0033] Rollins, R. C. , and L. Rüdenberg . 1977. “Chromosome Numbers of Cruciferae III.” Contributions From the Gray Herbarium of Harvard University 207, no. 1977: 101–116. 10.5962/p.336444.

[ece370523-bib-0034] Rousset, F. 2008. “GENEPOP'007: A Complete Re‐Implementation of the GENEPOP Software for Windows and Linux.” Molecular Ecology Resources 8, no. 1: 103–106. 10.1111/j.1471-8286.2007.01931.x. 21585727

[ece370523-bib-0035] Salywon, A. , and D. A. Dierig . 2006. “Isolation and Characterization of Microsatellite Loci in *Lesquerella fendleri* (Brassicaceae) and Cross‐Species Amplification.” Molecular Ecology Notes 6, no. 2: 382–384. 10.1111/j.1471-8286.2006.01241.x.

[ece370523-bib-0036] Salywon, A. M. , J. P. Rebman , and D. A. Dierig . 2022. “Documented Chromosome Number Determinations in Some *Physaria* Species (Brassicaceae).” Journal of the Botanical Research Institute of Texas 16, no. 2: 499–504. 10.17348/jbrit.v16.i2.1262.

[ece370523-bib-0037] Schuelke, M. 2000. “An Economic Method for the Fluorescent Labeling of PCR Fragments.” Nature Biotechnology 18: 233–234. 10.1038/72708. 10657137

[ece370523-bib-0038] Slatkin, M. 1987. “Gene Flow and the Geographic Structure of Natural Populations.” Science 236, no. 4803: 787–792. 10.1126/science.3576198. 3576198

[ece370523-bib-0039] Slatkin, M. 1993. “Isolation by Distance in Equilibrium and Non‐equilibrium Populations.” Evolution 47, no. 1: 264–279. 10.2307/2410134. 28568097

[ece370523-bib-0040] Tecic, D. L. , J. L. McBride , M. L. Bowles , and D. L. Nickrent . 1998. “Genetic Variability in the Federal Threatened mead's Milkweed, *Asclepias meadii* Torrey (Asclepiadaceae), as Determined by Allozyme Electrophoresis.” Annals of the Missouri Botanical Garden 85, no. 1: 97–109. 10.2307/2992000.

[ece370523-bib-0041] Tkach, K. , and M. J. Watson . 2023. “Publication and Use of Genetic Tools in Conservation Management Applications—A Systematic Review.” Journal of Applied Ecology 60, no. 8: 1522–1536. 10.1111/1365-2664.14433.

[ece370523-bib-0042] United States Fish and Wildlife Service . 2009. “Endangered and Threatened Wildlife and Plants; Partial 90‐Day Finding on a Petition to List 475 Species in the Southwestern United States as Threatened or Endangered With Critical Habitat.” Federal Register 74: 66866–66905.

[ece370523-bib-0043] USDA. United States Department of Agriculture . 2014. “Land and Resource Management Plan for the Kaibab National Forest; Coconino, Yavapai, and Mojave Counties, Arizona.” Forest Service Southwestern Region, MB‐R3‐07‐17.

[ece370523-bib-0044] Wallace, C. , J. Coons , and B. Molano‐Flores . 2021. “Pollen Grain Morphology of *Physaria kingii* subsp. *kaibabensis* and Two Congeners.” Southwestern Naturalist 65, no. 1: 63–66. 10.1894/0038-4909-65.1.63.

[ece370523-bib-0045] Whiteley, A. R. , S. W. Fitzpatrick , W. C. Funk , and D. A. Tallmon . 2015. “Genetic Rescue to the Rescue.” Trends in Ecology & Evolution 30, no. 1: 42–49. 10.1016/j.tree.2014.10.009. 25435267

[ece370523-bib-0046] Zaya, D. N. , S. A. Leicht‐Young , N. B. Pavlovic , C. S. Hetrea , and M. V. Ashley . 2017. “Mislabeling of an Invasive Vine (*Celastrus orbiculatus*) as a Native Congener (*C. scandens*) in Horticulture.” Invasive Plant Science and Management 10, no. 4: 313–321. 10.1017/inp.2017.37.

[ece370523-bib-0047] Zhou, Y. F. , R. J. Abbott , Z. Y. Jiang , F. K. Du , R. I. Milne , and J. Q. Liu . 2010. “Gene Flow and Species Delimitation: A Case Study of Two Pine Species With Overlapping Distributions in Southeast China.” Evolution 64, no. 8: 2342–2352. 10.1111/j.1558-5646.2010.00988.x. 20298431

