# Supplementary material for: Genetic Diversity and Structure of Physaria on the Kaibab Plateau: Implications for Conservation
Source: Ecol Evol. 2024 Nov 18;14(11):e70523. doi: 10.1002/ece3.70523 (PMC11570800; doi:10.1002/ece3.70523)

Supplementary Materials

Supplement 1. Microsatellite diversity of 463 *Physaria* samples (94 *P. arizonica* and 369 *P. kingii* samples) summarized by locus: number of different alleles (A), effective number of alleles (A_e), observed heterozygosity (H_O), expected heterozygosity (H_E), and fixation index (*F*).

Species	Locus	A	A_e	H_O	H_E	*F*
P. arizonica	LF6	3.333	2.150	0.472	0.476	0.019
	LF14	2.833	2.083	0.352	0.410	0.112
	LF20	3.167	1.541	0.355	0.355	-0.038
	LF24	3.833	2.753	0.221	0.332	0.451
	LF25	0.167	0.167	0.000	0.000	
	LF27	2.500	1.549	0.304	0.326	0.055
	LF29	5.667	3.646	0.831	0.722	-0.152
	LF35	3.333	2.001	0.436	0.404	-0.107
	PF8	2.833	2.340	0.402	0.382	-0.053
	PF33	3.000	2.198	0.485	0.425	-0.143
	PF37	2.333	1.627	0.328	0.325	-0.024
	PF62	3.333	2.237	0.461	0.536	0.134
	PF82	1.833	1.140	0.120	0.104	-0.098
P. kingii	LF6	2.050	1.246	0.159	0.175	0.078
	LF14	3.600	2.449	0.440	0.548	0.197
	LF20	3.150	2.290	0.501	0.538	0.094
	LF24	8.200	5.028	0.729	0.744	0.036
	LF25	6.150	4.104	0.661	0.737	0.112
	LF27	1.950	1.145	0.174	0.188	0.017
	LF29	2.150	1.264	0.182	0.179	-0.026
	LF35	4.450	2.871	0.577	0.622	0.077
	PF8	6.250	3.317	0.668	0.669	0.013
	PF33	3.400	2.283	0.533	0.509	-0.058
	PF37	5.600	2.388	0.506	0.553	0.067
	PF62	3.350	1.761	0.377	0.402	0.054
	PF82	2.850	2.019	0.480	0.470	-0.010

Supplement 2. Microsatellite diversity of 463 *Physaria* samples collected from 13 loci was summarized by locus for each population: number of samples (N), number of different alleles (A), effective number of alleles (A_e), observed heterozygosity (H_O), expected heterozygosity (H_E), and fixation index (*F*). Populations that show significant deviations from Hardy-Weinberg expectations are indicated by *.

Species	Sampling Location	Locus	N	A	A_e	H_O	H_E	*F*
P. arizonica	462	LF6	20	3	2.089	0.450	0.521	0.137
		LF14	20	2	1.882	0.250	0.469	0.467
		LF20	20	5	1.709	0.500	0.415	-0.205
		LF24	11	1	1.000	0.000	0.000	
		LF25	0	0	0.000	0.000	0.000	
		LF27	20	2	1.471	0.400	0.320	-0.250
		LF29	8	6	4.414	0.750	0.773	0.030
		LF35	15	6	2.711	0.733	0.631	-0.162
		PF8	7	6	4.900	0.714	0.796	0.103
		PF33	0	0	0.000	0.000	0.000	
		PF37	17	3	1.966	0.588	0.491	-0.197
		PF62	14	4	2.465	0.571	0.594	0.039
		PF82	19	3	1.520	0.421	0.342	-0.231
	HR	LF6	13	2	1.954	0.385	0.488	0.212
		LF14	20	5	4.372	0.650	0.771	0.157
		LF20	20	4	2.417	0.550	0.586	0.062
		LF24	19	14	9.757	0.789	0.898	0.120
		LF25	0	0	0.000	0.000	0.000	
		LF27	20	3	1.629	0.400	0.386	-0.036
		LF29	20	6	3.653	0.750	0.726	-0.033
		LF35	20	5	2.057	0.550	0.514	-0.071
		PF8	20	4	2.888	0.700	0.654	-0.071
		PF33	20	4	2.279	0.500	0.561	0.109
		PF37	20	3	1.107	0.100	0.096	-0.039
		PF62	20	5	2.346	0.450	0.574	0.216
		PF82	20	2	1.051	0.050	0.049	-0.026
	LeF	LF6	19	7	2.854	0.632	0.650	0.028
		LF14	16	5	1.969	0.563	0.492	-0.143
		LF20	17	4	1.360	0.294	0.265	-0.111
		LF24	18	1	1.000	0.000	0.000	
		LF25	0	0	0.000	0.000	0.000	
		LF27	19	4	1.851	0.474	0.460	-0.030
		LF29	20	7	3.065	0.700	0.674	-0.039
		LF35	0	0	0.000	0.000	0.000	

	PF8	0	0	0.000	0.000	0.000	
	PF33	7	6	4.667	0.857	0.786	-0.091
	PF37	19	2	1.819	0.263	0.450	0.415
	PF62	15	2	1.991	0.533	0.498	-0.071
	PF82	20	2	1.161	0.150	0.139	-0.081
LFR	LF6	10	3	2.410	0.700	0.585	-0.197
	LF14	7	1	1.000	0.000	0.000	
	LF20	0	0	0.000	0.000	0.000	
	LF24	6	2	1.385	0.000	0.278	1.000
	LF25	0	0	0.000	0.000	0.000	
	LF27	10	3	1.852	0.300	0.460	0.348
	LF29	8	6	3.200	0.875	0.688	-0.273
	LF35	4	5	4.571	0.750	0.781	0.040
	PF8	0	0	0.000	0.000	0.000	
	PF33	3	5	4.500	1.000	0.778	-0.286
	PF37	10	3	2.381	0.600	0.580	-0.034
	PF62	8	3	2.032	0.375	0.508	0.262
	PF82	10	2	1.105	0.100	0.095	-0.053
Nre	LF6	12	4	2.595	0.667	0.615	-0.085
	LF14	11	2	1.308	0.273	0.236	-0.158
	LF20	6	3	1.412	0.333	0.292	-0.143
	LF24	7	2	1.690	0.286	0.408	0.300
	LF25	1	1	1.000	0.000	0.000	
	LF27	12	2	1.492	0.250	0.330	0.242
	LF29	11	4	3.667	0.909	0.727	-0.250
	LF35	3	2	1.385	0.333	0.278	-0.200
	PF8	5	7	6.250	1.000	0.840	-0.190
	PF33	0	0	0.000	0.000	0.000	
	PF37	12	2	1.492	0.417	0.330	-0.263
	PF62	10	4	2.985	0.500	0.665	0.248
	PF82	12	1	1.000	0.000	0.000	
SPH	LF6	12	1	1.000	0.000	0.000	
	LF14	8	2	1.969	0.375	0.492	0.238
	LF20	11	3	2.350	0.455	0.574	0.209
	LF24	4	3	1.684	0.250	0.406	0.385
	LF25	0	0	0.000	0.000	0.000	
	LF27	12	1	1.000	0.000	0.000	
	LF29	8	5	3.879	1.000	0.742	-0.347
	LF35	4	2	1.280	0.250	0.219	-0.143
	PF8	0	0	0.000	0.000	0.000	
	PF33	9	3	1.742	0.556	0.426	-0.304

		PF37	12	1	1.000	0.000	0.000	
		PF62	12	2	1.600	0.333	0.375	0.111
		PF82	11	1	1.000	0.000	0.000	
P. kingii subsp. *kaibabensis*	758	LF6	20	2	1.105	0.000	0.095	1.000
		LF14	20	5	3.404	0.400	0.706	0.434
		LF20	20	4	3.065	0.600	0.674	0.109
		LF24	20	8	7.143	0.850	0.860	0.012
		LF25	20	7	4.969	0.750	0.799	0.061
		LF27	20	1	1.000	0.000	0.000	
		LF29	20	1	1.000	0.000	0.000	
		LF35	20	6	3.587	0.750	0.721	-0.040
		PF8	20	6	2.581	0.500	0.613	0.184
		PF33	19	3	1.925	0.684	0.481	-0.424
		PF37	19	4	2.292	0.474	0.564	0.160
		PF62	19	3	2.329	0.579	0.571	-0.015
		PF82	19	2	1.296	0.158	0.229	0.309
	AZT	LF6	19	2	1.819	0.368	0.450	0.182
		LF14	20	5	2.930	0.450	0.659	0.317
		LF20	20	3	2.133	0.550	0.531	-0.035
		LF24	20	11	6.504	0.900	0.846	-0.064
		LF25	20	5	4.061	0.550	0.754	0.270
		LF27	20	2	1.536	0.250	0.349	0.283
		LF29	20	5	1.449	0.350	0.310	-0.129
		LF35	20	4	3.292	0.550	0.696	0.210
		PF8	20	5	3.213	0.650	0.689	0.056
		PF33	20	2	1.995	0.550	0.499	-0.103
		PF37	20	9	2.395	0.400	0.583	0.313
		PF62	20	3	1.504	0.350	0.335	-0.045
		PF82	20	4	2.279	0.600	0.561	-0.069
	CIP	LF6	20	2	1.536	0.350	0.349	-0.004
		LF14	20	3	2.111	0.400	0.526	0.240
		LF20	20	2	1.923	0.600	0.480	-0.250
		LF24	19	12	8.699	1.000	0.885	-0.130
		LF25	20	8	4.651	0.750	0.785	0.045
		LF27	20	2	1.600	0.300	0.375	0.200
		LF29	20	1	1.000	0.000	0.000	
		LF35	20	6	4.145	0.850	0.759	-0.120
		PF8	19	5	2.516	0.579	0.602	0.039
		PF33	20	2	1.956	0.450	0.489	0.079
		PF37	19	7	2.560	0.421	0.609	0.309
		PF62	20	3	1.288	0.250	0.224	-0.117

	PF82	20	3	1.995	0.700	0.499	-0.404
CrL	LF6	20	3	1.354	0.300	0.261	-0.148
	LF14	20	4	2.067	0.500	0.516	0.031
	LF20	20	4	2.025	0.550	0.506	-0.086
	LF24	20	8	3.419	0.650	0.708	0.081
	LF25	20	7	3.279	0.650	0.695	0.065
	LF27	20	2	1.882	0.250	0.469	0.467
	LF29	20	2	1.105	0.100	0.095	-0.053
	LF35	20	6	3.960	0.750	0.748	-0.003
	PF8	20	6	3.200	0.650	0.688	0.055
	PF33	20	2	1.923	0.600	0.480	-0.250
	PF37	20	6	1.724	0.400	0.420	0.048
	PF62	20	3	1.288	0.250	0.224	-0.117
	PF82	20	3	2.462	0.600	0.594	-0.011
DPt	LF6	20	2	1.724	0.300	0.420	0.286
	LF14	20	4	2.640	0.600	0.621	0.034
	LF20	20	4	2.658	0.750	0.624	-0.202
	LF24	20	11	5.517	0.950	0.819	-0.160
	LF25	20	6	3.902	0.700	0.744	0.059
	LF27	20	2	1.161	0.150	0.139	-0.081
	LF29	20	3	1.559	0.350	0.359	0.024
	LF35*	20	5	3.980	0.200	0.749	0.733
	PF8	20	4	3.306	0.800	0.698	-0.147
	PF33	20	4	1.441	0.350	0.306	-0.143
	PF37	20	5	2.374	0.550	0.579	0.050
	PF62	20	2	1.406	0.250	0.289	0.134
	PF82	20	2	1.923	0.500	0.480	-0.042
DrL	LF6	20	2	1.406	0.250	0.289	0.134
	LF14	20	5	3.333	0.550	0.700	0.214
	LF20	20	4	1.878	0.400	0.468	0.144
	LF24	20	9	3.960	0.700	0.748	0.064
	LF25	20	6	4.301	0.600	0.768	0.218
	LF27	20	1	1.000	0.000	0.000	
	LF29	20	2	1.536	0.450	0.349	-0.290
	LF35*	19	4	2.551	0.158	0.608	0.740
	PF8	20	8	3.604	0.750	0.723	-0.038
	PF33	19	3	2.215	0.684	0.548	-0.247
	PF37	20	6	1.843	0.300	0.458	0.344
	PF62	20	3	1.354	0.200	0.261	0.234
	PF82	20	2	1.600	0.400	0.375	-0.067
Hen	LF6	20	2	1.280	0.050	0.219	0.771

	LF14	20	3	2.198	0.350	0.545	0.358
	LF20	20	4	2.204	0.450	0.546	0.176
	LF24	20	7	4.020	0.750	0.751	0.002
	LF25	20	6	3.361	0.650	0.703	0.075
	LF27	20	2	1.406	0.350	0.289	-0.212
	LF29	20	3	1.421	0.350	0.296	-0.181
	LF35	20	4	3.347	0.600	0.701	0.144
	PF8	20	9	5.128	0.900	0.805	-0.118
	PF33	20	3	2.241	0.600	0.554	-0.084
	PF37	20	6	2.952	0.600	0.661	0.093
	PF62	20	3	1.766	0.350	0.434	0.193
	PF82	20	3	1.766	0.500	0.434	-0.153
KbL	LF6	20	2	1.051	0.050	0.049	-0.026
	LF14	20	3	2.556	0.600	0.609	0.014
	LF20	20	3	2.740	0.600	0.635	0.055
	LF24	20	5	3.865	0.700	0.741	0.056
	LF25	20	4	2.241	0.450	0.554	0.187
	LF27	20	1	1.000	0.000	0.000	
	LF29	20	2	1.536	0.250	0.349	0.283
	LF35	20	4	1.839	0.300	0.456	0.342
	PF8	20	7	5.263	0.800	0.810	0.012
	PF33	20	2	1.980	0.300	0.495	0.394
	PF37	20	6	2.417	0.750	0.586	-0.279
	PF62	20	3	1.985	0.650	0.496	-0.310
	PF82	20	2	1.600	0.300	0.375	0.200
MV	LF6	20	2	1.105	0.100	0.095	-0.053
	LF14	20	4	2.367	0.550	0.578	0.048
	LF20	20	3	2.266	0.550	0.559	0.016
	LF24	20	9	3.433	0.450	0.709	0.365
	LF25	20	8	5.926	0.850	0.831	-0.023
	LF27	20	3	1.512	0.350	0.339	-0.033
	LF29	20	2	1.051	0.050	0.049	-0.026
	LF35	20	4	2.749	0.650	0.636	-0.022
	PF8	20	9	3.252	0.800	0.693	-0.155
	PF33	20	4	1.597	0.450	0.374	-0.204
	PF37	20	6	2.500	0.650	0.600	-0.083
	PF62	20	2	1.051	0.050	0.049	-0.026
	PF82	20	3	2.180	0.500	0.541	0.076
Pvy	LF6	20	3	1.164	0.150	0.141	-0.062
	LF14	20	3	2.360	0.650	0.576	-0.128
	LF20	20	3	2.216	0.700	0.549	-0.276

		LF24	20	8	6.154	0.900	0.838	-0.075
		LF25	18	7	4.500	0.833	0.778	-0.071
		LF27	20	2	1.161	0.150	0.139	-0.081
		LF29	20	4	1.677	0.400	0.404	0.009
		LF35	20	6	3.306	0.500	0.698	0.283
		PF8	19	5	3.840	0.842	0.740	-0.139
		PF33	19	3	1.875	0.474	0.467	-0.015
		PF37	20	7	1.636	0.450	0.389	-0.158
		PF62	20	3	1.361	0.300	0.265	-0.132
		PF82	20	2	2.000	0.300	0.500	0.400
	TL	LF6	20	2	1.105	0.100	0.095	-0.053
		LF14	20	4	2.192	0.550	0.544	-0.011
		LF20	20	3	2.299	0.450	0.565	0.204
		LF24	20	9	6.897	0.800	0.855	0.064
		LF25	19	6	2.542	0.526	0.607	0.132
		LF27	20	2	1.220	0.200	0.180	-0.111
		LF29	20	3	1.597	0.450	0.374	-0.204
		LF35	20	5	3.030	0.550	0.670	0.179
		PF8	18	8	3.289	0.556	0.696	0.202
		PF33	19	2	1.362	0.316	0.266	-0.187
		PF37	20	5	1.896	0.450	0.473	0.048
		PF62	20	6	2.041	0.300	0.510	0.412
		PF82	20	4	1.839	0.400	0.456	0.123
	VTL	LF6	19	2	1.111	0.105	0.100	-0.056
		LF14	20	4	2.299	0.600	0.565	-0.062
		LF20	20	3	2.930	0.650	0.659	0.013
		LF24	20	11	5.298	0.750	0.811	0.076
		LF25	19	7	5.641	0.789	0.823	0.040
		LF27	20	2	1.051	0.050	0.049	-0.026
		LF29	20	3	1.597	0.300	0.374	0.197
		LF35	20	4	2.540	0.500	0.606	0.175
		PF8	20	8	4.000	0.750	0.750	0.000
		PF33	20	3	2.694	0.750	0.629	-0.193
		PF37	20	5	1.914	0.550	0.478	-0.152
		PF62	20	3	2.089	0.500	0.521	0.041
		PF82	20	2	1.956	0.550	0.489	-0.125
P. kingii subsp. *latifolia*	429	LF6	20	2	1.051	0.050	0.049	-0.026
		LF14	20	5	3.883	0.650	0.743	0.125
		LF20	20	3	2.730	0.650	0.634	-0.026
		LF24	20	9	6.250	0.850	0.840	-0.012
		LF25	20	8	5.970	0.650	0.833	0.219

	LF27	20	3	1.164	0.150	0.141	-0.062
	LF29	20	1	1.000	0.000	0.000	
	LF35	20	4	2.388	0.700	0.581	-0.204
	PF8	20	8	3.419	0.800	0.708	-0.131
	PF33	20	4	2.388	0.650	0.581	-0.118
	PF37	20	6	2.477	0.600	0.596	-0.006
	PF62	20	5	2.180	0.550	0.541	-0.016
	PF82	20	4	1.806	0.500	0.446	-0.120
DPk	LF6	20	3	1.164	0.150	0.141	-0.062
	LF14	20	4	3.524	0.550	0.716	0.232
	LF20	20	3	2.974	0.550	0.664	0.171
	LF24	20	9	5.674	0.750	0.824	0.090
	LF25	20	6	2.712	0.650	0.631	-0.030
	LF27	20	3	1.164	0.150	0.141	-0.062
	LF29	20	2	1.161	0.150	0.139	-0.081
	LF35	20	5	2.703	0.750	0.630	-0.190
	PF8	20	10	4.040	0.700	0.753	0.070
	PF33	20	3	2.122	0.450	0.529	0.149
	PF37	20	6	3.846	0.850	0.740	-0.149
	PF62	19	3	1.885	0.632	0.470	-0.345
	PF82	20	4	2.210	0.500	0.548	0.087
Lk3	LF6	20	2	1.161	0.150	0.139	-0.081
	LF14	20	5	2.500	0.550	0.600	0.083
	LF20	20	4	3.137	0.750	0.681	-0.101
	LF24	20	10	6.250	0.750	0.840	0.107
	LF25	20	6	4.233	0.800	0.764	-0.047
	LF27	20	3	1.946	0.350	0.486	0.280
	LF29	20	2	1.342	0.200	0.255	0.216
	LF35	20	6	3.008	0.700	0.668	-0.049
	PF8	19	6	3.820	0.789	0.738	-0.069
	PF33	20	3	2.180	0.400	0.541	0.261
	PF37	20	5	1.713	0.450	0.416	-0.081
	PF62	20	4	1.790	0.400	0.441	0.093
	PF82	20	3	1.946	0.500	0.486	-0.028
LP	LF6	20	2	1.105	0.100	0.095	-0.053
	LF14	0	0	0.000	0.000	0.000	
	LF20	20	3	2.346	0.250	0.574	0.564
	LF24	0	0	0.000	0.000	0.000	
	LF25	17	7	5.161	0.824	0.806	-0.021
	LF27	18	4	1.815	0.556	0.449	-0.237
	LF29	20	1	1.000	0.000	0.000	

	LF35	6	4	3.130	0.833	0.681	-0.224
	PF8	11	2	1.862	0.545	0.463	-0.179
	PF33	19	2	1.232	0.211	0.188	-0.118
	PF37	4	2	1.600	0.500	0.375	-0.333
	PF62	16	3	2.124	0.563	0.529	-0.063
	PF82	19	2	1.296	0.158	0.229	0.309
SHO	LF6	13	1	1.000	0.000	0.000	
	LF14	12	5	3.349	0.583	0.701	0.168
	LF20	13	3	1.733	0.308	0.423	0.273
	LF24	13	9	6.898	0.923	0.855	-0.080
	LF25	12	6	4.645	0.833	0.785	-0.062
	LF27	13	4	1.271	0.231	0.213	-0.083
	LF29	13	1	1.000	0.000	0.000	
	LF35	12	4	2.717	0.917	0.632	-0.451
	PF8	13	8	3.841	0.846	0.740	-0.144
	PF33	13	3	2.209	0.462	0.547	0.157
	PF37	12	5	1.895	0.500	0.472	-0.059
	PF62	13	5	2.139	0.462	0.533	0.133
	PF82	13	2	1.954	0.692	0.488	-0.418
MOC	LF6	10	2	1.342	0.300	0.255	-0.176
	LF14	11	2	2.000	0.273	0.500	0.455
	LF20	11	2	1.308	0.091	0.236	0.614
	LF24	11	9	5.042	0.909	0.802	-0.134
	LF25	11	4	3.143	0.455	0.682	0.333
	LF27	0	0	0.000	0.000	0.000	
	LF29	11	1	1.000	0.000	0.000	
	LF35	11	3	1.847	0.455	0.459	0.009
	PF8	11	3	2.068	0.091	0.517	0.824
	PF33	11	7	2.916	0.818	0.657	-0.245
	PF37	8	4	2.612	0.125	0.617	0.797
	PF62	11	2	1.424	0.182	0.298	0.389
	PF82	11	3	2.350	0.636	0.574	-0.108
PgL	LF6	10	2	1.342	0.300	0.255	-0.176
	LF14*	10	3	2.273	0.000	0.560	1.000
	LF20	10	2	1.600	0.100	0.375	0.733
	LF24	9	7	3.857	0.778	0.741	-0.050
	LF25	10	5	3.846	0.900	0.740	-0.216
	LF27	0	0	0.000	0.000	0.000	
	LF29	10	2	1.105	0.100	0.095	-0.053
	LF35	10	2	1.980	0.700	0.495	-0.414
	PF8	10	3	1.504	0.300	0.335	0.104

	PF33	10	5	3.774	0.600	0.735	0.184
	PF37	10	4	3.333	0.300	0.700	0.571
	PF62	10	4	2.198	0.600	0.545	-0.101
	PF82	10	5	4.444	0.700	0.775	0.097
LCY	LF6	13	1	1.000	0.000	0.000	
	LF14	15	1	1.000	0.000	0.000	
	LF20	15	3	1.625	0.467	0.384	-0.214
	LF24	14	3	1.682	0.214	0.406	0.472
	LF25	6	4	3.000	0.000	0.667	1.000
	LF27	0	0	0.000	0.000	0.000	
	LF29	15	2	1.142	0.133	0.124	-0.071
	LF35	15	3	1.312	0.133	0.238	0.439
	PF8	14	5	2.596	0.714	0.615	-0.162
	PF33	15	8	5.625	0.867	0.822	-0.054
	PF37	15	8	3.782	0.800	0.736	-0.088
	PF62	15	4	2.018	0.133	0.504	0.736
	PF82	15	2	1.471	0.400	0.320	-0.250

Supplement 3. Pairwise genetic divergence for the 26 *Physaria* populations shown in F_{ST} (lower diagonal) and G'_{ST} (upper diagonal) values. Populations of different taxonomic units were labeled with different colors: *P. arizonica* (white), *P. kingii* subsp. *kaibabensis* (light gray), and *P. kingii* subsp. *latifolia* (dark gray). Darker red cells indicate greater values for the differentiation metrics.

	462	HR	LeF	LFR	Nre	SPH	758	AZT	CIP	CrL	DPt	DrL	Hen	KbL	MV	Pvy	TL	VTL	429	DPk	Lk3	LP	SHO	MOC	PgL	LCY
462	--	0.21	0.10	0.05	0.11	0.38	0.45	0.39	0.44	0.42	0.43	0.45	0.43	0.46	0.47	0.46	0.43	0.45	0.45	0.42	0.41	0.46	0.47	0.44	0.42	0.50
HR	0.21	--	0.23	0.22	0.23	0.43	0.44	0.38	0.41	0.41	0.42	0.43	0.41	0.42	0.46	0.43	0.42	0.42	0.42	0.40	0.39	0.52	0.44	0.43	0.38	0.48
LeF	0.10	0.23	--	0.16	0.15	0.28	0.50	0.42	0.47	0.46	0.49	0.49	0.48	0.50	0.52	0.50	0.48	0.48	0.48	0.46	0.45	0.53	0.50	0.48	0.42	0.55
LFR	0.05	0.22	0.16	--	0.09	0.41	0.47	0.40	0.44	0.44	0.45	0.46	0.43	0.47	0.48	0.46	0.46	0.45	0.45	0.43	0.43	0.49	0.45	0.41	0.38	0.54
Nre	0.10	0.23	0.15	0.09	--	0.42	0.46	0.39	0.42	0.41	0.43	0.44	0.42	0.44	0.48	0.45	0.45	0.44	0.44	0.45	0.42	0.46	0.47	0.46	0.45	0.49
SPH	0.37	0.41	0.29	0.42	0.42	--	0.60	0.55	0.58	0.58	0.58	0.60	0.58	0.60	0.61	0.60	0.59	0.59	0.58	0.56	0.57	0.67	0.60	0.57	0.50	0.62
758	0.46	0.44	0.51	0.48	0.47	0.58	--	0.08	0.11	0.14	0.07	0.13	0.08	0.13	0.11	0.09	0.06	0.10	0.08	0.03	0.06	0.12	0.11	0.35	0.31	0.46
AZT	0.39	0.38	0.43	0.40	0.39	0.52	0.08	--	0.00	0.05	0.06	0.04	0.06	0.10	0.08	0.05	0.05	0.09	0.07	0.05	0.03	0.15	0.08	0.33	0.28	0.44
CIP	0.44	0.42	0.48	0.45	0.44	0.56	0.11	0.00	--	0.04	0.07	0.06	0.07	0.11	0.07	0.07	0.06	0.10	0.06	0.08	0.04	0.14	0.07	0.35	0.29	0.47
CrL	0.42	0.41	0.47	0.45	0.42	0.56	0.14	0.05	0.04	--	0.11	0.07	0.09	0.16	0.14	0.08	0.10	0.14	0.12	0.11	0.03	0.19	0.13	0.40	0.35	0.49
DPt	0.43	0.42	0.50	0.45	0.43	0.56	0.07	0.06	0.07	0.11	--	0.09	0.05	0.12	0.08	0.07	0.05	0.10	0.09	0.06	0.07	0.14	0.09	0.34	0.28	0.46
DrL	0.45	0.43	0.49	0.47	0.46	0.58	0.13	0.04	0.06	0.07	0.09	--	0.11	0.10	0.11	0.07	0.07	0.12	0.12	0.08	0.06	0.19	0.11	0.37	0.32	0.48
Hen	0.44	0.41	0.48	0.44	0.43	0.56	0.08	0.06	0.07	0.09	0.05	0.11	--	0.10	0.09	0.03	0.06	0.09	0.08	0.07	0.05	0.16	0.10	0.35	0.31	0.47
KbL	0.46	0.43	0.50	0.49	0.46	0.59	0.13	0.10	0.11	0.16	0.12	0.10	0.10	--	0.13	0.11	0.12	0.13	0.10	0.10	0.11	0.20	0.13	0.38	0.33	0.48
MV	0.48	0.47	0.52	0.50	0.49	0.60	0.11	0.08	0.07	0.14	0.08	0.11	0.09	0.13	--	0.08	0.07	0.09	0.04	0.08	0.09	0.16	0.04	0.36	0.29	0.51
Pvy	0.46	0.43	0.51	0.47	0.46	0.58	0.09	0.05	0.07	0.08	0.07	0.07	0.03	0.11	0.08	--	0.06	0.07	0.07	0.08	0.03	0.18	0.09	0.38	0.32	0.48
TL	0.43	0.42	0.49	0.47	0.44	0.58	0.06	0.05	0.06	0.10	0.05	0.07	0.06	0.12	0.07	0.06	--	0.11	0.08	0.04	0.04	0.12	0.08	0.37	0.31	0.48
VTL	0.45	0.42	0.48	0.46	0.46	0.57	0.10	0.09	0.10	0.14	0.10	0.12	0.09	0.13	0.09	0.07	0.11	--	0.05	0.10	0.09	0.18	0.07	0.33	0.29	0.45
429	0.45	0.43	0.48	0.46	0.45	0.57	0.08	0.07	0.06	0.12	0.09	0.12	0.08	0.10	0.04	0.07	0.08	0.05	--	0.07	0.07	0.16	0.01	0.32	0.26	0.46
DPk	0.42	0.40	0.46	0.44	0.43	0.54	0.03	0.05	0.08	0.11	0.06	0.08	0.07	0.10	0.08	0.08	0.04	0.10	0.07	--	0.05	0.14	0.09	0.33	0.28	0.45
Lk3	0.41	0.39	0.45	0.43	0.41	0.55	0.06	0.03	0.04	0.03	0.07	0.06	0.05	0.11	0.09	0.03	0.04	0.09	0.07	0.05	--	0.14	0.08	0.35	0.31	0.46
LP	0.47	0.53	0.54	0.52	0.50	0.68	0.12	0.14	0.13	0.19	0.13	0.19	0.15	0.20	0.16	0.18	0.12	0.18	0.15	0.14	0.14	--	0.21	0.43	0.37	0.47
SHO	0.47	0.44	0.51	0.46	0.47	0.59	0.11	0.08	0.07	0.13	0.09	0.11	0.10	0.13	0.04	0.09	0.08	0.07	0.01	0.09	0.08	0.21	--	0.31	0.26	0.49
MOC	0.45	0.43	0.49	0.42	0.47	0.57	0.35	0.33	0.35	0.40	0.34	0.37	0.35	0.38	0.37	0.38	0.37	0.33	0.32	0.32	0.35	0.43	0.32	--	0.09	0.46
PgL	0.43	0.38	0.43	0.38	0.45	0.49	0.31	0.28	0.30	0.36	0.28	0.32	0.31	0.33	0.29	0.32	0.32	0.29	0.26	0.28	0.31	0.38	0.26	0.09	--	0.45
LCY	0.51	0.48	0.54	0.56	0.55	0.63	0.45	0.43	0.47	0.49	0.46	0.47	0.46	0.48	0.51	0.48	0.47	0.45	0.46	0.45	0.45	0.47	0.49	0.46	0.46	--

Supplement 4a. The Delta K plot for the STRUCTURE analysis for all *Physaria* samples.

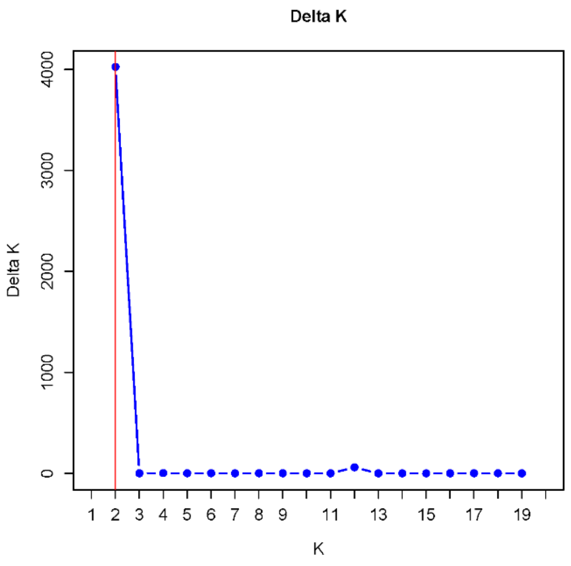

Supplement 4b. The Med K plots for the STRUCTURE analysis for all *Physaria* samples.

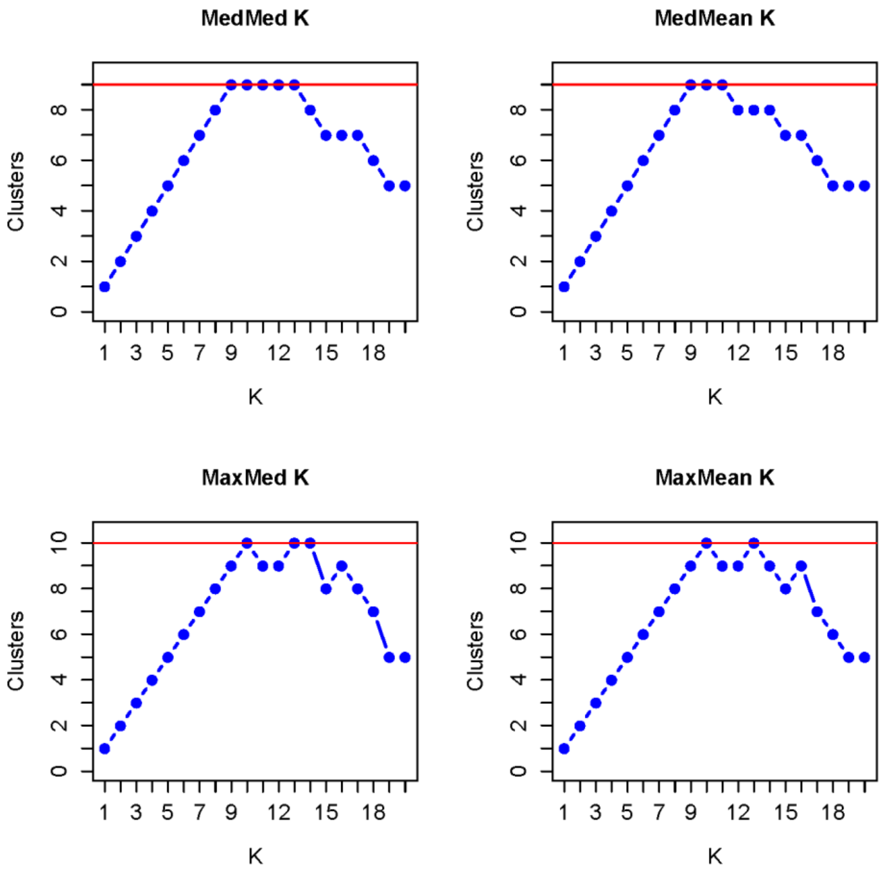

Supplement 4c. Bar plot results from STRUCTURE for all *Physaria* samples (n = 463). Each column represents the genetic assignment of a single individual. These are the results for the model with K = 3 clusters. Asterisks indicate populations off of the Kaibab Plateau.

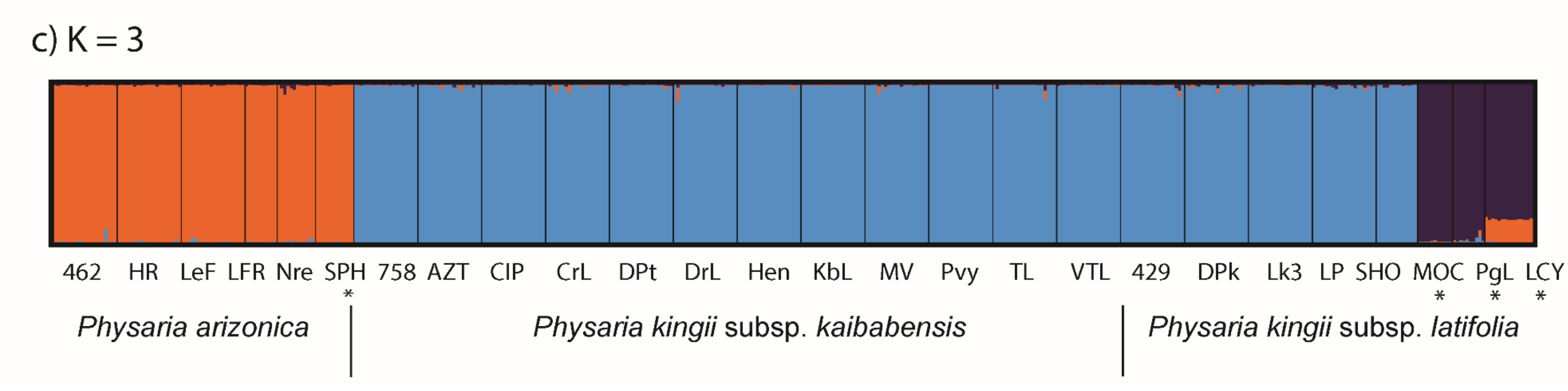

Supplement 4d. Bar plot results from STRUCTURE for all *Physaria* samples (n = 463). Each column represents the genetic assignment of a single individual. These are the results for the model with K = 4 clusters. Asterisks indicate populations off of the Kaibab Plateau.

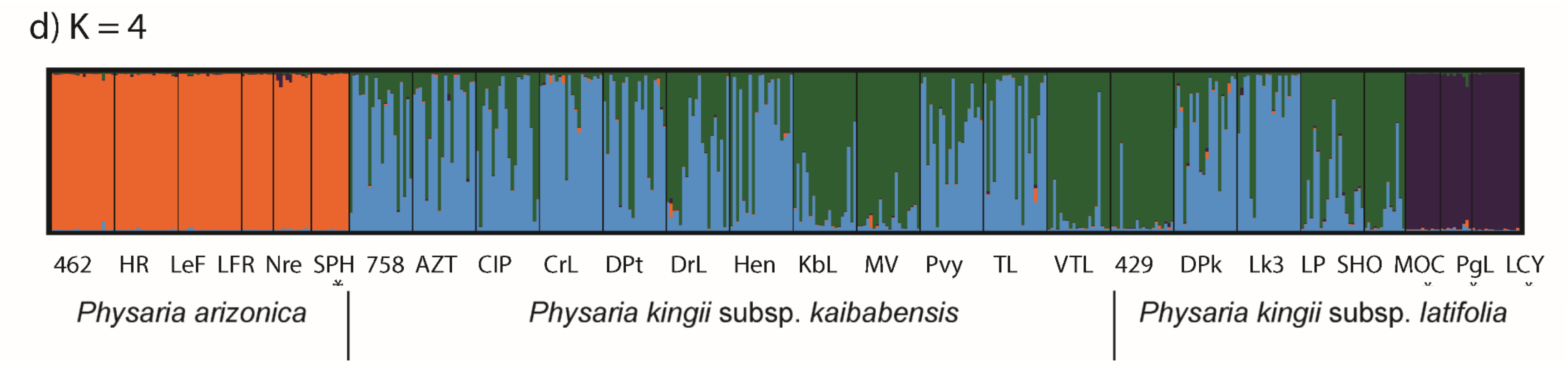

Supplement 5a. The Delta K plot for the STRUCTURE analysis for *P. kingii* samples.

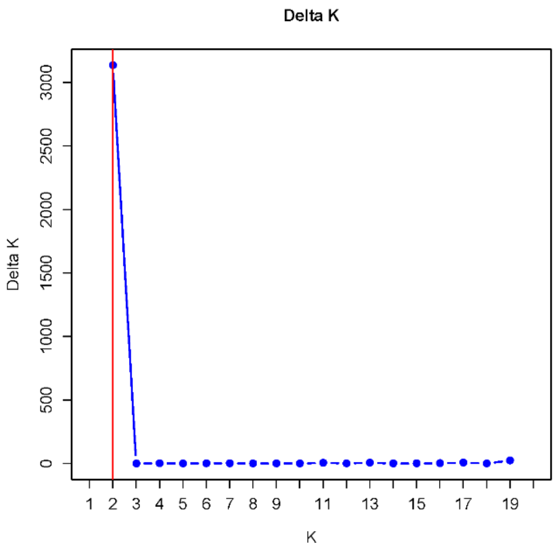

Supplement 5b. The Med K plots for the STRUCTURE analysis for *P. kingii* samples.

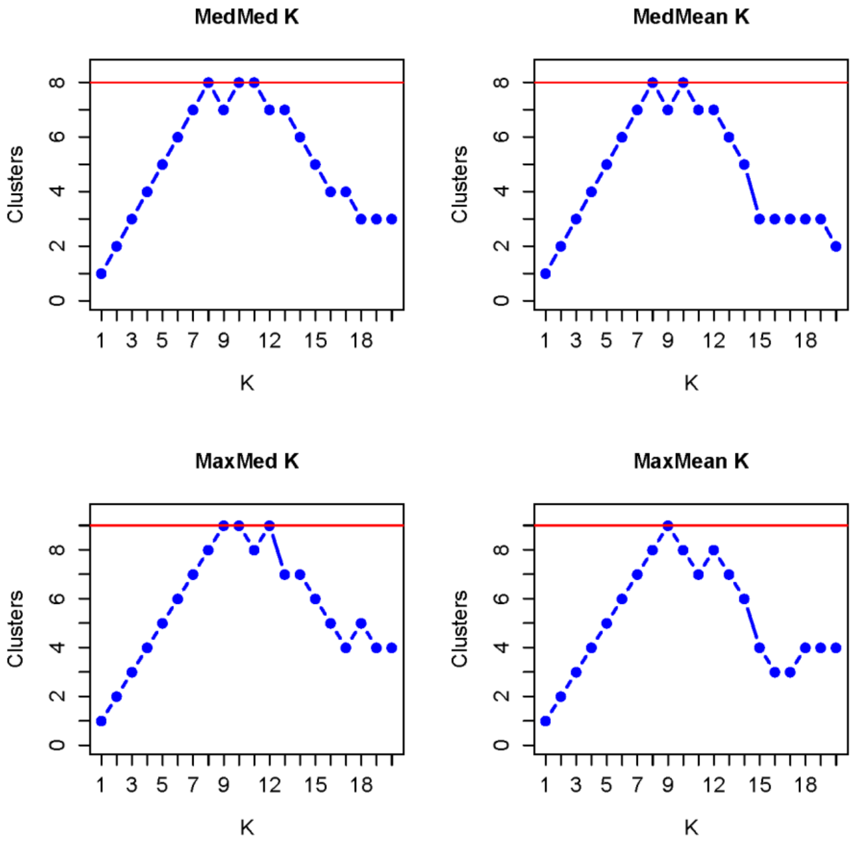

Supplement 6a. The Delta K plot for the STRUCTURE analysis for *P. arizonica* samples.

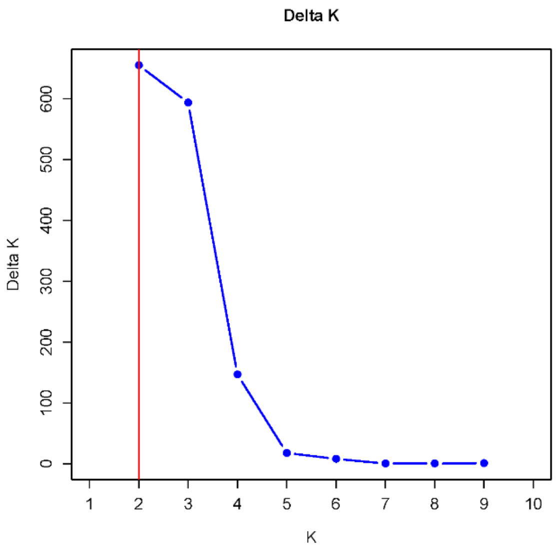

Supplement 6b. The Med K plots for the STRUCTURE analysis for *P. arizonica* samples.

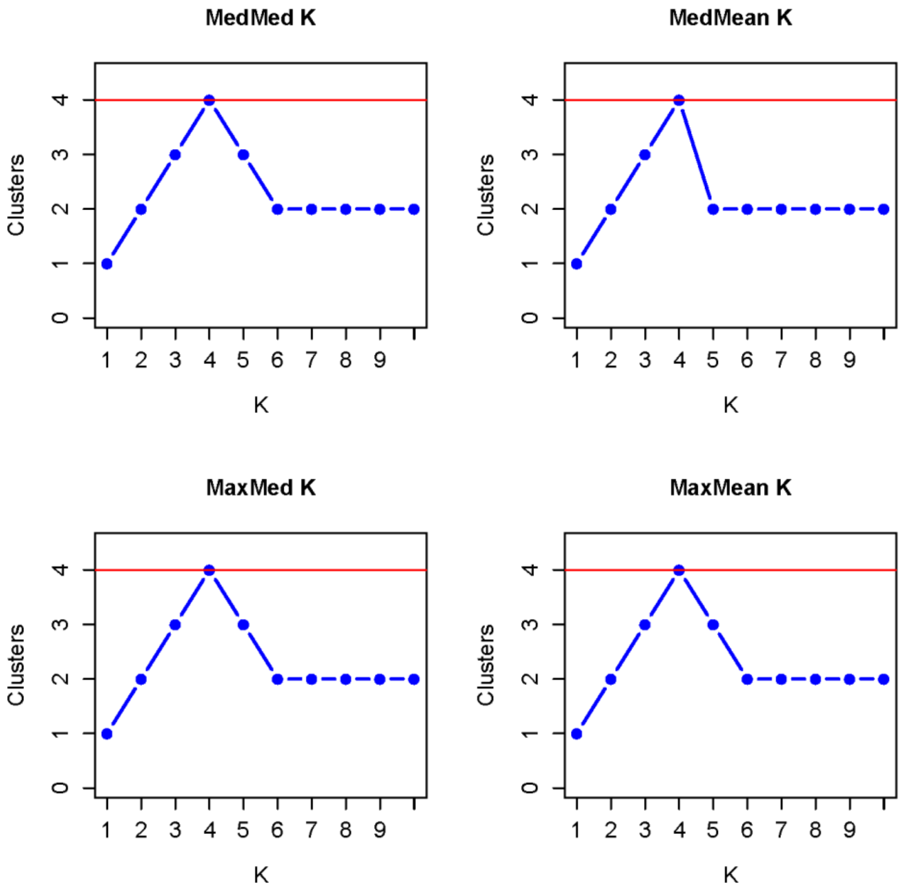

Supplement: Supplementary file 1 — Data S1 [file ECE3-14-e70523-s001.pdf]
